# Motor Neuron Disease Systematic Multi-Arm Adaptive Randomised Trial (MND-SMART): a multi-arm, multi-stage, adaptive, platform, phase III randomised, double-blind, placebo-controlled trial of repurposed drugs in motor neuron disease

**DOI:** 10.1136/bmjopen-2022-064173

**Published:** 2022-07-07

**Authors:** Charis Wong, Rachel S Dakin, Jill Williamson, Judith Newton, Michelle Steven, Shuna Colville, Maria Stavrou, Jenna M Gregory, Elizabeth Elliott, Arpan R Mehta, Jeremy Chataway, Robert J Swingler, Richard Anthony Parker, Christopher J Weir, Nigel Stallard, Mahesh K B Parmar, Malcolm R Macleod, Suvankar Pal, Siddharthan Chandran

**Affiliations:** 1 Centre of Clinical Brain Sciences, University of Edinburgh, Edinburgh, UK; 2 Euan MacDonald Centre for Motor Neuron Disease Research, University of Edinburgh, Edinburgh, UK; 3 Anne Rowling Regenerative Neurology Clinic, University of Edinburgh, Edinburgh, UK; 4 Edinburgh Clinical Trials Unit, Usher Institute of Population Health Sciences and Informatics, University of Edinburgh, Edinburgh, UK; 5 UK Dementia Research Institute Edinburgh, University of Edinburgh, Edinburgh, UK; 6 Institute of Medical Sciences, University of Aberdeen, Aberdeen, UK; 7 Queen Square Multiple Sclerosis Centre, Department of Neuroinflammation, UCL Queen Square Institute of Neurology, Faculty of Brain Sciences, University College London, London, UK; 8 National Institute for Health Research, University College London Hospitals, Biomedical Research Centre, London, UK; 9 Medical Research Council Clinical Trials Unit at UCL, Institute of Clinical Trials and Methodology, University College London, London, UK; 10 London North West University Healthcare NHS Trust, Northwick Park Hospital, London, UK; 11 Statistics and Epidemiology, Division of Health Sciences, Warwick Medical School, University of Warwick, Coventry, UK

**Keywords:** Motor neurone disease, Clinical trials, Neurology, STATISTICS & RESEARCH METHODS

## Abstract

**Introduction:**

Motor neuron disease (MND) is a rapidly fatal neurodegenerative disease. Despite decades of research and clinical trials there remains no cure and only one globally approved drug, riluzole, which prolongs survival by 2–3 months. Recent improved mechanistic understanding of MND heralds a new translational era with many potential targets being identified that are ripe for clinical trials. Motor Neuron Disease Systematic Multi-Arm Adaptive Randomised Trial (MND-SMART) aims to evaluate the efficacy of drugs efficiently and definitively in a multi-arm, multi-stage, adaptive trial. The first two drugs selected for evaluation in MND-SMART are trazodone and memantine.

**Methods and analysis:**

Initially, up to 531 participants (177/arm) will be randomised 1:1:1 to oral liquid trazodone, memantine and placebo. The coprimary outcome measures are the Amyotrophic Lateral Sclerosis Functional Rating Scale Revised (ALSFRS-R) and survival. Comparisons will be conducted in four stages. The decision to continue randomising to arms after each stage will be made by the Trial Steering Committee who receive recommendations from the Independent Data Monitoring Committee. The primary analysis of ALSFRS-R will be conducted when 150 participants/arm, excluding long survivors, have completed 18 months of treatment; if positive the survival effect will be inferentially analysed when 113 deaths have been observed in the placebo group. The trial design ensures that other promising drugs can be added for evaluation in planned trial adaptations. Using this novel trial design reduces time, cost and number of participants required to definitively (phase III) evaluate drugs and reduces exposure of participants to potentially ineffective treatments.

**Ethics and dissemination:**

MND-SMART was approved by the West of Scotland Research Ethics Committee on 2 October 2019. (REC reference: 19/WS/0123) Results of the study will be submitted for publication in a peer-reviewed journal and a summary provided to participants.

**Trial registration numbers:**

European Clinical Trials Registry (2019-000099-41); NCT04302870.

**Strengths and limitations of this study**

- The Motor Neuron Disease-Systematic Multi-Arm Adaptive Randomised Trial is the first multi-arm multi-stage (MAMS) phase III trial in motor neuron disease to launch in Europe.
- This novel trial design aims to achieve efficiencies in time, cost and requirements for sample size through planned trial adaptations: ineffective arms can be dropped at interim analysis while new arms can be added.
- Broad inclusion criteria allow wider participation and ensures generalisability of results in a heterogenous condition.
- Drugs are selected using a combined approach of a data-driven framework incorporating published literature and expert opinion.
- The complexities of the conduct of this MAMS trial necessitate particular operational considerations.

## Introduction

Motor neuron disease (MND) is an incurable, rapidly progressive and invariably fatal disease characterised by selective motor neuron loss. Despite 125 phase II to III drug trials between 2008 and 2019, riluzole, licensed in the UK by the National Institute for Health and Care Excellence in 2001, remains the only disease-modifying drug with marketing authorisation in the UK and Europe, with a median survival benefit of 2–3 months.^1 2^ Across the UK historical MND trial participation is less than 5%.^2^ Recently, edaravone and masitinib have emerged as promising candidates following positive trials in enriched trial populations.^3 4^ Edaravone was licensed in Japan and the USA in 2015 and 2017, respectively. However, evidence of generalisable survival benefit for these drugs remain limited; in a long-term prospective cohort study, edaravone did not improve survival or slow functional decline.^5^

There are many challenges facing clinical trials for MND ranging from comparatively blunt outcome measures to disease factors including rapid progression and heterogeneity. Benchmark outcomes for MND trials are largely ordinal clinical rating scales and survival.^2 6^ To detect a significant treatment effect using these measures, large sample sizes are often needed to achieve adequate study power, especially when using traditional fixed two-arm designs. To account for heterogeneity, many previous trials used strict inclusion criteria.^2 7^ However, a mean exclusion rate of 60% in 38 previous trials did not reduce heterogeneity in survival or functional decline.^7^ Strict inclusion criteria, diagnostic delay and the rapidly progressive and fatal nature of MND often results in a short time window for people with MND (pwMND) to participate in trials. Furthermore, trial retention is often poor—in a recent review, 40% of trials reported attrition rates ≥20%.^2^ Adherence with trial protocol often becomes challenging with increasing disability. Movement, speech, swallowing and respiration are commonly affected, the latter causing some pwMND to be dependent on enteral feeding and ventilatory support. Cognitive and behavioural changes also occur in approximately 35% of pwMND with up to 15% having frontotemporal dementia (FTD).^8 9^ Against this background, there have been major advances in our understanding of the pathobiology of MND as well as an increasing number of candidate medicines being identified supporting innovation in trial design for MND.

Multi-arm multi-stage (MAMS) and other adaptive platform trials have been used successfully in other fields, especially cancer medicine and infectious diseases, and are particularly effective where there are several promising medicines without clear a priori evidence to favour one over the other.^2 10 11^ A multi-arm trial allows simultaneous evaluation of multiple treatments against a single control group. An adaptive trial design allows additional arms to be added in what is in effect a ‘continuous’ trial platform. These features can provide major efficiency gains in time, cost and sample size requirements compared with running multiple (and almost certainly consecutive) conventional two-arm studies, each with their own control arm. Beside Motor Neuron Disease-Systematic Multi-Arm Adaptive Randomised Trial (MND-SMART) trial, there are two other declared adaptive platform trials in MND: US-based HEALEY amyotrophic lateral sclerosis Platform Trial^12^ (ClinicalTrials.gov registration number NCT04297683) and European-based Treatment and Research Initiative to Cure amyotrophic lateral sclerosis ‘MAGNET’ platform trial.^13^

Drug repurposing has proven successful in many areas, including multiple sclerosis.^14^ The lower development cost and shorter development timelines with availability of long-term safety data makes this a particularly attractive option for devastating conditions like MND where trial attrition rates are historically high.^2^ Rapid advances in genomics, molecular and human stem cell technology has led to better understanding of the underlying pathobiology of MND and discovery of novel drug targets. Computational advances have made living systematic reviews of the entire published literature of MND and other neurodegenerative diseases which may share similar pathways feasible, thus providing a robust, comprehensive and continually updated evidence-base to identify, evaluate and prioritise candidate drugs for trials.^15–17^ These principles were used to inform drug selection for the Multiple Sclerosis-Secondary Progressive Multi-Arm Randomisation Trial (MS-SMART), a multi-arm trial of repurposed drugs in progressive multiple sclerosis.^18 19^ The initial candidate drugs for MS-SMART based on this approach were ibudilast, amiloride and riluzole, but due to drug supply issue, ibudilast was switched to fluoxetine. Although the drugs tested in MS-SMART did not show clear evidence of activity, ibudilast slowed progression of brain atrophy in a separate phase II trial.^20^

Against this background, MND-SMART is a phase III randomised MAMS trial of repurposed drugs in MND. The first two candidate drugs to be tested are trazodone and memantine.

## Methods and analysis

### Trial objectives

The primary objective of MND-SMART is to assess the clinical effects of putative neuroprotective drugs compared with placebo as measured by rate of change in score of the Amyotrophic Lateral Sclerosis Functional Rating Scale Revised (ALSFRS-R), and conditional on identifying benefit on this, to assess the effect of these drugs on survival. Secondary objectives are to assess the safety profile of candidate drugs, and their effects on time to King’s stage 4a (nutritional failure), time to King’s stage 4b (respiratory failure), cognitive function and behaviour as assessed using the Edinburgh Cognitive and Behavioural ALS Screen (ECAS), respiratory function measured by forced vital capacity (FVC), anxiety and depression measured by the Hospital Anxiety and Depression Scale (HADS) and quality of life evaluation using EQ-5D-5L.

### Overview of design

**Figure 1:**
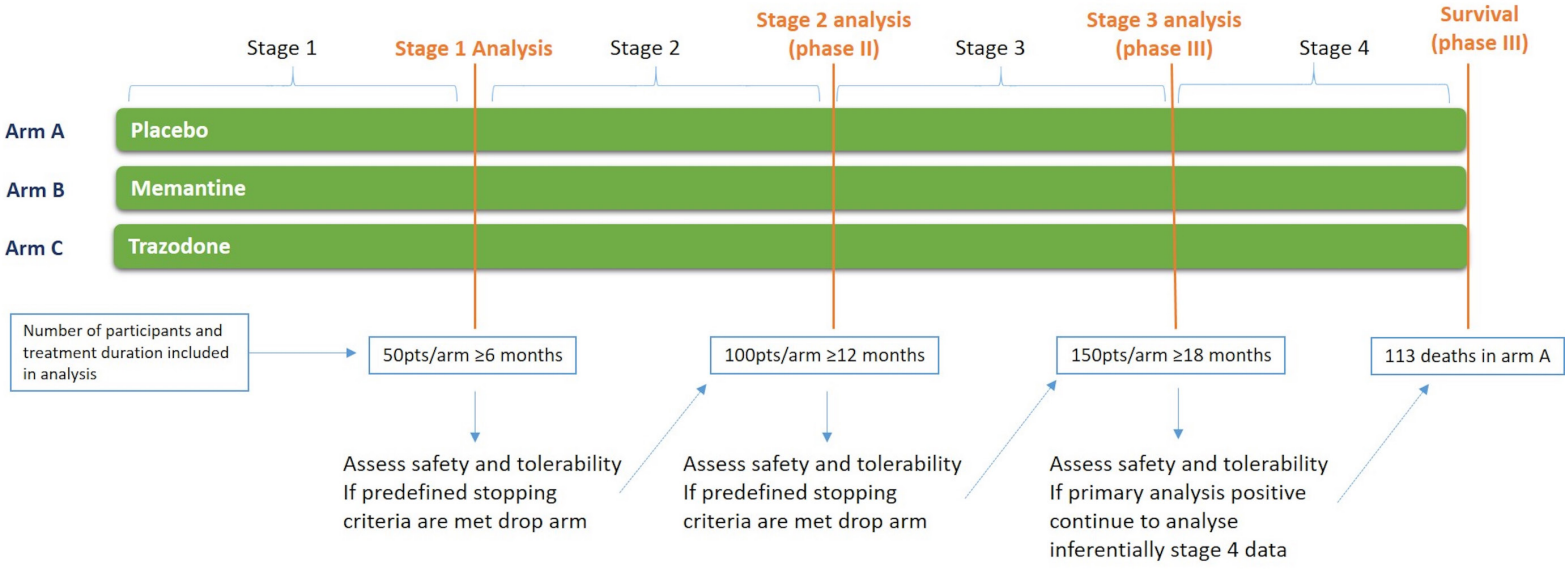
Trial overview of Motor Neuron Disease Systematic Multi-Arm Adaptive Randomised Trial.

**Figure 2:**
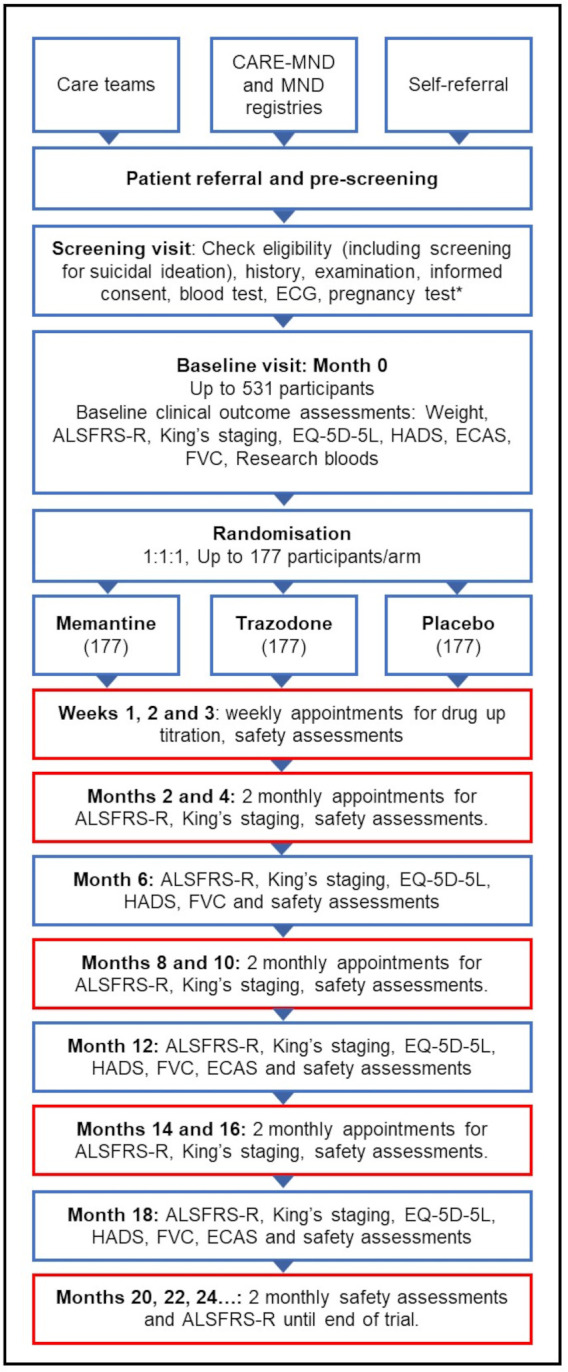
Motor Neuron Disease Systematic Multi-Arm Adaptive Randomised Trial participant timeline. Boxes with red outline indicate study visits which can be conducted by telephone or secure videoconferencing. ALSFRS-R, Amyotrophic Lateral Sclerosis Functional Rating Scale Revised; CARE-MND, Clinical Audit Research Evaluation Motor Neuron Disease; ECAS, Edinburgh Cognitive and Behavioural ALS Screen; FVC, forced vital capacity; HADS, Hospital Anxiety and Depression Scale. *for women of childbearing potential.

MND-SMART is a phase III double-blind, randomised, placebo-controlled MAMS platform trial. MND-SMART will initially assess the efficacy of memantine (arm A) and trazodone (arm B) against a single contemporaneous placebo control arm (arm C) with investigational medicinal products (IMPs) delivered as liquids. Participants are randomised 1:1:1 to arms A, B and C with up to 531 pwMND (177/arm) randomised to complete the analysis of these arms (figure 1). Allowing for early withdrawal and long survivors, this will give 150 participants/group in the primary analysis of ALSFRS-R. Patients will have five appointments in the first four to 8 weeks followed by assessments every 2 months, most completed remotely, and two appointments for treatment cessation when this is required (figure 2).

The ALSFRS-R comparisons will be conducted in three stages (figure 1) with the opportunity to stop randomisation to arms that do not meet the predefined continuing criteria (table 1) at the end of stages 1 of 2. Stage 1 will be completed when 50 participants/arm (excluding long survivors defined as >8 years since diagnosis at baseline) have completed 6 months of treatment and stage 2 will be completed when 100 participants per arm (excluding long survivors) have completed 12 months of treatment. The primary analysis (stage 3) will be conducted based on rate of decline of ALSFRS-R when 150 participants/arm (excluding long survivors) complete 18 months of treatment. Participants will continue to take their allocated treatment until 113 deaths have been observed in the placebo group, or stage 3 analysis data are collected—whichever is later, with the primary analysis of survival being performed at this point.

**Table 1:** Timing and guidance for stage 1 and stage 2 analyses

Stage	Number of pwALS/arm excluding long survivors	Criterion to continue to next stage
Stage 1	50 with ≥6 months of follow-up	95% CI of the rate of change in ALSFRS-R compared with placebo must include a relative improvement of 25% in the rate of decline
Stage 2	100 with ≥12 months of follow-up	Improvement in rate of change in ALSFRS-R compared with placebo significant at pairwise one-sided 10% level

ALSFRS-R, Amyotrophic Lateral Sclerosis Functional Rating Scale Revised; pwALS, people with ALS.

The study protocol follows the Standard Protocol Items: Recommendations for Interventional Trials guidelines^21^ and was registered on the European Clinical Trials Registry on 25 July 2019 and ClinicalTrials.gov on 10 March 2020.

### Planned trial adaptations

In addition to stopping arms that do not meet continuing criteria at the end of stage 1 and 2, we intend to introduce new arms in the future by substantial amendment. Any new arm will be compared with contemporaneous controls with each comparison following the multi-stage process described earlier. As long as treatments are distinct, for example belonging to different classes of medicine, no multiplicity adjustments will be made in the analysis of pairwise comparisons.^22–24^

### Re-entry of participants into the trial

Participants will be able to enter the trial on more than once occasion, under certain conditions. To ensure a participant does not contribute data to more than one arm in the same treatment comparison, they will only be able to re-enter MND-SMART if they satisfy one of the following criteria: the arm they were randomised to has been dropped; they are in the control group and all experimental arms open at the time at which they were randomised have been dropped; or their data have been included in a final survival-based comparison. Any participant re-entering the trial will be screened for eligibility in the same way as a trial naïve individual. Previous trial entry will be noted to ensure their medical and treatment history is linked to allow for long-term follow-up of potential signals and understanding of investigational drugs exposed to.

### Participants, interventions and outcomes

#### Trial setting

MND-SMART is recruiting across the UK with 17 recruiting centres (listed on mnd-smart.org) at 10 March 2022. Research staff will enter data on site via a secure electronic case report form (eCRF), with quality control and analysis conducted independently by Edinburgh Clinical Trials Unit (ECTU).

#### Eligibility criteria

Eligibility criteria for MND-SMART are shown in table 2.

**Table 2:** MND-SMART eligibility criteria (protocol version 6.0)

Inclusion criteria	
Confirmed diagnosis of MND (including the following subtypes: amyotrophic lateral sclerosis by El Escorial criteria (possible, probable and definite), primary lateral sclerosis and progressive muscular atrophy). Over 18 years of age. Women of childbearing potential must have a negative pregnancy test within 7 days prior to, or at, the baseline visit.	Women of childbearing potential and fertile men must be using an appropriate method of contraception to avoid any unlikely teratogenic effects of the selected drugs from time of consent, to 4 weeks after treatment inclusive. Willing and able to comply with the trial protocol and ability to understand and complete questionnaires. Written informed consent (this can be signed by a proxy in the case of limb dysfunction).
Exclusion criteria	
Diagnosis of frontotemporal dementia (FTD-MND) or any other significant psychiatric disorder that prevents informed consent being given. Patients in the manic phase of bipolar disorder. Alcoholism (self-reported). Active suicide ideation assessed using the Columbia-Suicide Severity Rating Scale. On concurrent investigational medication (including biological therapy). Known hypersensitivity, including hereditary fructose intolerance, or adverse reaction to the active substances and their excipients or any medical history contraindicating use of any of the investigational medicinal products (IMPs). Pregnancy or breastfeeding females. If ALT, ALP, bilirubin or GGT>3 times the upper limit of normal; creatinine clearance (creatinine clearance or eGFR)<30 mL/min; or serum free T4>25 pmol/L or TSH<0.2 mU/L.	If corrected QT interval on 12 lead ECG>450 ms. Diagnosis of ventricular arrhythmias, heart block or in the immediate recovery period after myocardial infarction (<6 weeks). Already taking any of the IMPs in the protocol. Contraindications to any of the IMPs according to summary of product characteristics (SPC). Taking a medication that interacts with the active substances and their excipients according to the SPCs, including but not limited to; dextromethorphan, amantadine; ketamine, monoamine-oxidase inhibitors ((MAOIs): rasagiline, selegiline, safinamide, tranylcypromine, phenelzine, isocarboxazid, moclobemide). Patients who the PI considers will not be able to comply with the study protocol.

ALP, alkaline phosphatase; ALT, alanine aminotransferase; eGFR, estimated glomerular filtration rate; GGT, gamma-glutamyltransferase; MND, motor neuron disease; MND-SMART, Motor Neuron Disease Systematic Multi-Arm Adaptive Randomised Trial; PI, Principal Investigator; TSH, thyroid-stimulating hormone.

#### Ascertainment and recruitment

Potential participants are identified through self-referral from potential participants who may be made aware of the trial by the media, literature or website (www.mnd-smart.org), by members of clinical and care teams, and using the existing National MND Care/Research platform (CARE-MND) in Scotland and other UK wide MND registries that contain contact details of people who have consented to be contacted directly about relevant research opportunities. Potential participants will be informed about the study and given a patient information sheet. They can be prescreened by the individual’s clinical team or, in Scotland, against trial criteria using information from CARE-MND.

The screening visit includes documentation of written informed consent. If a participant is physically unable to give written consent due to limb dysfunction, verbal consent can be given with a witness independent of the trial signing the consent form. A clinical trial physician will evaluate each potential participant against the trial eligibility criteria and recruit participants. Each centre is required to maintain an anonymised log of all ineligible patients and eligible patients who declined participation.

The first participant was randomised on 27 February 2020.

Identifying the research interventions for priority evaluation in MND-SMART.

We conducted a two-stage systematic review of the clinical literature of MND and four other neurodegenerative diseases (Alzheimer’s disease, Parkinson’s disease, Huntington’s disease and Multiple Sclerosis) which may share similar pathways, and of the preclinical MND literature to inform identification and selection of investigational drugs for MND-SMART by an expert panel.^25^ Through this structured process memantine and trazodone were selected for evaluation. Memantine is a non-competitive N-methyl-D-aspartate receptor antagonist used in the management of Alzheimer’s disease. It was shown to significantly delay disease progression and improve survival in mouse models carrying a high copy number of SOD1^G93A^.^26^ Three early phase MND clinical trials using memantine showed some evidence of safety and potential signals for efficacy in ALSFRS-R and in cerebrospinal and neurophysiological biomarkers.^27–29^ Trazodone is an atypical antidepressant drug of the serotonin antagonist and reuptake inhibitor class. In an unbiased drug screen, trazodone inhibited Protein Kinase RNA-like endoplasmic reticulum kinase (PERK), which is pivotal to stress granule formation, a common feature of neurodegenerative diseases.^30^ Inhibition of PERK was found to be beneficial in an ALS fly model as well as in an in vitro neuronal assay of TDP-43 injury.^31^ Modulation of ER-stress response by trazodone resulted in improvement in survival of prion disease and FTD animal models.^30^ In Huntington’s disease mouse models, trazodone improved behavioural outcomes and exerted mitochondrial protective effects,^32^ a pathway of interest in MND.^33^ In a phase II trial in 31 people with FTD, trazodone improved cognition as assessed by the neuropsychiatric inventory.^34^

#### Drug allocation, titration and monitoring

Participants are randomised to memantine hydrochloride 10 mg/5 mL oral solution, trazodone hydrochloride 100 mg/5 mL oral solution or placebo oral solution containing no active pharmaceutical ingredient via a web-based randomisation system to maintain allocation concealment. Drugs and placebo are manufactured by Huddersfield Pharmacy Specials as liquids with identical colour, flavour and consistency. Weekly, during the first 4 weeks of the study, a clinical trial physician assesses participants for adverse events (AEs) and tolerability. IMP dose is also titrated using the dosing schedule shown in table 3. Participants are asked to record adherence, drug doses and any reasons for non-adherence.

**Table 3:** Motor Neuron Disease Systematic Multi-Arm Adaptive Randomised Trial dose titration schedule

	Baseline (week 0) ‘quarter dose’: 2.5 mL	Week 1 ‘half dose’: 5 mL	Week 2 ‘three quarter dose’: 7.5 mL	Week 3 ‘full dose’: 10 mL
Memantine	5 mg	10 mg	15 mg	20 mg
Trazodone	50 mg	100 mg	150 mg	200 mg
Placebo	2.5 mL	5 mL	7.5 mL	10 mL

Participants may stop treatment early with appropriate down-titration due to unacceptable AEs, pregnancy, inter-current illness that prevents further treatment, and any changes in the participant’s condition justifying discontinuation of treatment according to investigators’ discretion, including as part of end-of-life care.

#### Concomitant care and interventions

All concomitant medications are permitted except dextromethorphan, amantadine, ketamine, rasagiline, selegiline, safinamide, tranylcypromine, phenelzine, isocarboxazid and moclobemide. Participants are allowed to start standard of care, including riluzole and interventions like gastrostomy and non-invasive ventilation (NIV), after randomisation.

### Outcome measures

#### Primary outcome measures

ALSFRS-R and survival are coprimary outcomes in MND-SMART. ALSFRS-R assesses MND-related disability in gross and fine motor tasks, bulbar function and respiratory status.^35^ This scale has been widely used and validated as an appropriate and accurate assessment of functional ability and disease progression. It is easy to administer and has been validated for administration in person or over the telephone or by videoconferencing with high inter-rater and intra-rater reliability.^36^

#### Secondary outcomes measures

Secondary outcomes for MND-SMART include time to King’s stage 4a and time to King’s stage 4b, cognitive function and behaviour as assessed using ECAS, FVC, HADS and EQ-5D-5L quality of life evaluation.

#### Exploratory outcome

An exploratory outcome investigating ALSFRS-R scores post 18 months participation is also included.

#### Safety and tolerability outcomes

All study visits will capture data on AEs and adherence.

### Participant timeline

Participant timeline and visit schedule is shown in figure 2 and table 4. Most study visits can be conducted by telephone or secure videoconferencing.

**Table 4:** MND-SMART visit and assessment schedule

Appointment	Time	Informed consent	Inclusion/exclusion review	Eligibility confirmation	Demography	Disease history	Medical history	Current medications	Safety bloods	ECG	Suicide ideation (CSSRS)	Pregnancy test	Randomisation	FVC	ALSFRS-R and King’s staging*	EQ-5D-5L	HADS	ECAS†	Substudies	Research bloods	Drug supply	Titration up	Compliance check	Adverse events
1 Screening		x	x	x	x	x	x	x	x	x	x	x‡							x					x
2 Baseline	Time 0		x	x				x				x§	x	x	x	x	x	x	x	x	x	¶		x
3, 4, 5**	Weeks 1, 2 and 3							x														x	x	x
6, 7, 9, 10, 12, 13**	Months 2, 4, 8, 10, 14, 16							x							x				x		x		x	x
8††	Month 6							x						x	x	x	x		x	x	x		x	x
11††	Month 12							x						x	x	x	x	x	x	x	x		x	x
14††	Month 18							x						x	x	x	x	x	x	x	x		x	x
15, 16, 17 …**	Months 20, 22, 24 …							x							x						x		x	x

*Modified King’s staging will take place at remote appointments.

†This assessment includes an interview with a partner/relative/friend/carer if the participant has consented. If an arm is dropped the investigational medicinal product (IMP) dose should be halved at the next scheduled appointment and participants then complete the two treatment cessation appointments. Appointments from 15 onwards are the same to be repeated every 2 months.

‡For women of childbearing potential.

§If more than 7 days from previous test.

¶Start IMP.

**These appointments can be completed using video conferencing or telephone call.

††These appointments should be completed face to face wherever possible.

ALSFRS-R, Amyotrophic Lateral Sclerosis Functional Rating Scale Revised; CSSRS, Columbia-Suicide Severity Rating Scale; ECAS, Edinburgh Cognitive and Behavioural ALS Screen; FVC, forced vital capacity; HADS, Hospital Anxiety and Depression Scale; MND-SMART, Motor Neuron Disease-Systematic Multi-Arm Adaptive Randomised Trial.

### Number of patients required

For sample size calculations, simulation studies were performed using data from Pooled Resource Open-Access ALS Clinical Trials Open Access ALS Clinical Trial database which pooled data from several MND clinical trials.^37 38^ The simulations included interim analyses at the end of stages 1 and 2. For arms continuing beyond the second stage, the primary analysis was simulated based on 18-month follow-up data for 150 participants/arm excluding long survivors. This primary analysis plan is described in the Statistical methods section, and it should be noted that the design is calculated controlling the pairwise error rate at 2.5% (one-sided). To allow for a drop-out rate of approximately 10%, and for the exclusion from the analysis of long survivors (estimated to make up 5% of the trial population), we will randomise 177 participants to each treatment arm (total 531).

The simulations demonstrated that for a true 25% reduction in the rate of decline of ALSFRS-R, the sample size is sufficient to give a probability of 86% of continuing beyond the stage 2 analysis into the final stage and a probability of continuing to the final stage and obtaining a significant result of 83%, equivalent to an independent phase III trial power of 90%. The probability of dropping an active arm at the first interim analysis is 23% if the true treatment effect is identical to the control group and 2% if there is a true 25% reduction in the average decrease in ALSFRS-R in that arm.

With regards to the survival coprimary outcome, follow-up will continue until 113 deaths have been observed in the placebo arm. With 113 deaths/arm, this test will have 90% power to detect an HR of 0.65. To maintain overall type I error control at 2.5% (one-sided), survival will be analysed inferentially for an IMP only if statistically significant benefit has been demonstrated on ALSFRS-R. In the situation that the ALSFRS-R outcome measure does not produce a statistically significant result overall survival data will be analysed to provide supporting information but will not provide pivotal evidence of efficacy.

### Assignment of interventions

#### Treatment allocation and blinding

Randomisation is performed at baseline visit via a secure web-based randomisation system employing the following minimisation variables: riluzole use, use of NIV and/or gastrostomy, and long survival (>8 years from diagnosis). The allocation sequence incorporates a random element based on computer-generated pseudo-random numbers to maintain unpredictability. Participants, investigators, care providers and outcome assessors are blinded to treatment allocation. To maintain blinding, each bottle is identified by a unique bottle number. In situations where medical management necessitates knowledge of the study medication, emergency unblinding can be done through the web-based randomisation system.

### Data collection, management and analysis

#### Data collection and management

Data are collected by trained site staff and entered into the eCRF via a web-based portal. Access to the eCRF is role-based and delegated site staff are assigned unique usernames. Activity on the eCRF is tracked and auditable. The trial database includes in-built measures to reduce data entry errors and ensure data quality. Regular central data checks are performed with queries and follow-up undertaken on a site by site basis. The eCRF is hosted by the University of Edinburgh in their secure environment as detailed in the trial data management plan.

#### Statistical methods

A Consolidated Standards of Reporting Trials flow diagram will be reported. Descriptive statistics will be used to summarise baseline characteristics for each treatment group. Participants will be included in the analysis if they were not long survivors at baseline and have at least one ALSFRS-R score recorded either at baseline or during follow-up.

A hierarchical normal linear model will be used to compare the rate of decrease in ALSFRS-R over the follow-up period in each of the two active treatment group arms with the placebo arm. The analysis will adjust for the effects of baseline minimisation variables. Long survivors at baseline are not included in the primary ALSFRS-R analysis and so adjustment for this minimisation variable is not required. Estimates and 95% CIs will be obtained for the difference in ALSFRS-R decrease for each active treatment group minus placebo. At the first interim analysis, each experimental arm will continue to the second stage if the upper limit of its 95% CI for the rate of change of ALSFRS-R compared with placebo lies below a 25% reduction in the rate of decline. For example, a 95% CI for reduction in rate of decline of (−10%, 30%) would lead to continuation of the experimental arm, whereas the interval (−20%, 15%) would result in discontinuation.

At the second interim analysis, if a significant treatment benefit relative to the placebo is observed at the pairwise two-sided 20% significance level, then the treatment will proceed to the final (end of stage 3) analysis. At the final analysis, each pairwise comparison of trazodone and memantine with placebo, will be tested using a one-sided 2.5% significance. Most types of intercurrent events will be handled in the estimand using the treatment policy strategy, except for deaths which will be handled using the hypothetical strategy. A detailed statistical analysis plan will contain full information on the estimands for ALSFRS-R and survival.

Analysis of the survival coprimary outcome at the end of stage 4 will be based on all participants, including long survivors at baseline. Kaplan-Meier statistics will estimate the survival function and the median survival will be calculated for each group with 95% CIs. Log-rank tests will be used to compare treatment and placebo survival curves and treatment effects will be estimated by a Cox proportional hazard model including covariates to allow for the effects of the baseline minimisation variables.

Preplanned subgroup analyses of primary and secondary outcome measures will be performed for: participants with different survival at baseline (>8 years, <8 years); participants with different subtypes of MND (amyotrophic lateral sclerosis, primary lateral sclerosis and progressive muscular atrophy); participants with C9orf72 expansions; participants with bulbar-onset and limb-onset forms of MND; participants age <40 or age >80; participants receiving support with NIV at randomisation and participants receiving feeding support with gastrostomy at randomisation.

Missing data handling methods will be described in the full statistical analysis plan.

### Monitoring

#### Data monitoring

An Independent Data Monitoring Committee (IDMC) oversees the safety of participants in the trial and review interim analyses. The committee regularly monitors safety data and makes recommendations on whether the trial should continue as planned and on the dropping of treatment arms following formal interim analyses. The committee is composed of independent members, without competing interests and meetings are conducted in accordance with the IDMC charter.

#### Adverse events

##### Detection, recording, and reporting AEs

Participants are asked about the occurrence of AEs at every study appointment. AE data are also available from participant diaries and laboratory results. Each AE will be assessed at site for seriousness, causality and severity. A central unblinded team assesses the expectedness of all adverse reactions and serious adverse reactions. AEs due to pre-existing medical conditions are only reported if medically judged to be an unexpected worsening during the study. AEs related to MND of a participant should not be recorded for the trial if consistent with disease progression.

##### Withdrawal of study participants and early termination of trial

Participants are free to withdraw from the study at any point or a participant can be withdrawn by investigators. If withdrawal occurs, the primary reason will be documented in the participant’s case record form. Discontinuing study drug does not constitute withdrawal—all attempts should be made to follow-up the participant as per protocol and/or to re-commence treatment (if discontinuation was due to AEs which have subsequently resolved). Participants can choose to stop all appointments but remain in the trial for follow-up of survival from medical records only. Withdrawn subjects will not be replaced. The IDMC can request trial arm suspension or termination, for example due to unacceptable AEs.

#### Auditing

University of Edinburgh and NHS Lothian Academic and Clinical Central Office for Research and Development (ACCORD) is the trial sponsor. ACCORD personnel or designees, will perform monitoring activities and study audits in accordance with the trial monitoring plan. Data are continually audited by the central team for return rate and completeness.

#### Patient and public involvement

Patient public involvement (PPI) has been crucial throughout the development of MND-SMART. Through support groups and a PPI advisory group, pwMND, their families and carers were consulted and provided suggestions to optimise trial recruitment and retention. These included limiting number of appointments or travel to appointments, use of remote appointments supported by telehealth, delivery of IMP to participants’ homes where necessary, use of liquid IMP formulations to enable participants with swallowing difficulties including those using gastrostomy to participate and remain in trial, and using broad inclusion criteria where the main exclusions are limited to contraindications to study drugs and lack of capacity to consent.

## Ethics and dissemination

### Research ethics approval

MND-SMART was approved by the West of Scotland Research Ethics Committee on 2 October 2019 (REC reference: 19/WS/0123).

### Protocol amendments

Protocol versions and substantial amendments to date are summarised in table 5.

**Table 5:** Protocol versions and substantial amendments to date

Protocol update	Protocol version	Protocol date	Reason for amendment
NA	V1	11 July 2019	N/A
Substantial amendment	V2*	20 August 2019	Addition of Clinical Trials Coordination and Facilitation Group (CTFG) definition of women of childbearing potential; and information on highly effective contraceptive methods. Additional pregnancy test added at the end of systemic exposure for women of childbearing potential
Substantial amendment—SA01	V3	24 October 2019	Clarification on trial design: Treatment and follow-up duration will continue until 200 patients/group have 18 months of follow-up (whichever is later) Guidance on prioritising primary endpoints, completing modified King’s staging in remote appointments and timescale for completing carer part of the ECAS, within 2 weeks of participant part of assessment. Addition that adverse events that are consistent with pre-existing medical conditions and/or the progression of MND do not need to be recorded in the CRF or reported (if serious) but should be noted in medical records. Confirmation of the procedure for assigning expectedness to adverse reactions by the central expectedness team.
Substantial amendment—SA02	V3	24 October 2019	Addition of site
Substantial amendment—SA03	V3	24 October 2019	Addition of sites
Substantial amendment—SA04	V3	24 October 2019	Change of PI
Substantial amendment—SA05	V5	1 March 2021	Updates to exclusion criteria. Confirmation on prescreening process and addition of remote consent using electronic software. Update to allow use of clinical results for eligibility review and outcome assessments and addition of possibility for CI to unblind. Confirmation patients who withdraw can consent to follow-up of survival from medical records. Addition of ability to cross cover for sites and updates to requirements to courier drugs direct to participants including removing need to return IMP bottles to site for compliance. Addition of sending patient-reported outcome questionnaires direct by email and clarification on assessments which can be performed remotely.
Substantial amendment—SA06	V5	1 March 2021	Addition of sites
Substantial amendment—SA07	V6	1 November 2021	Update to sample size based on move from family wise to pair wise type I error control. Removal of SNIP (sniff nasal inspiratory pressure) as a secondary outcome measure. Confirmation removal of IMPs from trial does not constitute study end, new IMPs will be added. Addition of exploratory objective to investigate ALSFRS-R post 18 months participation. Clarifications on consent for patients with limb dysfunction, coenrolment, IMP discontinuation and other points.

*Protocol version 2.0 was given CTA.

ALSFRS-R, Amyotrophic Lateral Sclerosis Functional Rating Scale Revised; CRF, case report form; CTA, Clinical Trial Authorisation; ECAS, Edinburgh Cognitive and Behavioural ALS Screen; IMP, investigational medicinal product; MND, motor neuron disease; PI, Principal Investigator.

### Confidentiality and access to data

All laboratory specimens, evaluation forms, reports and other records are identified with as little personal information as possible to maintain participant confidentiality. Access to personal information is restricted to individuals from the research team treating the participants, representatives of the Sponsor(s) and regulatory authorities. Those analysing samples or data will not have access to personal information. The trial master file is held by the Anne Rowling Clinic and the database is held by ECTU for a minimum of 25 years from the defined end of study point.

### Dissemination policy

To ensure data transparency, the results of any closed comparisons will be published in a peer-reviewed journal as soon as it is possible when the integrity of the trial will not be affected.^39^ Result of all analyses will be published. To maintain the scientific integrity of the study, data will not be released prior to the first publication of the results of the primary endpoint analysis, without the permission of the Trial Steering Committee (TSC). The TSC will agree a publication plan and must be consulted prior to release or publication of any study data. A summary of results will be provided to all participants via newsletters and the trial website.

## Supplementary Material

Reviewer comments

Author's manuscript
