# Supplementary material for: Motor Neuron Disease Systematic Multi-Arm Adaptive Randomised Trial (MND-SMART): a multi-arm, multi-stage, adaptive, platform, phase III randomised, double-blind, placebo-controlled trial of repurposed drugs in motor neuron disease
Source: BMJ Open. 2022 Jul 7;12(7):e064173. doi: 10.1136/bmjopen-2022-064173 (PMC9263927; doi:10.1136/bmjopen-2022-064173)
Supplement: Author's manuscript [file bmjopen-2022-064173.draft_revisions.pdf]

BMJ Open

BMJ Open is committed to open peer review. As part of this commitment we make the peer review history of every article we publish publicly available.

When an article is published we post the peer reviewers' comments and the authors' responses online. We also post the versions of the paper that were used during peer review. These are the versions that the peer review comments apply to.

The versions of the paper that follow are the versions that were submitted during the peer review process. They are not the versions of record or the final published versions. They should not be cited or distributed as the published version of this manuscript.

BMJ Open is an open access journal and the full, final, typeset and author-corrected version of record of the manuscript is available on our site with no access controls, subscription charges or pay-per-view fees (http://bmjopen.bmj.com).

If you have any questions on BMJ Open's open peer review process please email info.bmjopen@bmj.com

BMJ Open

Motor Neuron Disease Systematic Multi-Arm Adaptive Randomised Trial (MND-SMART): A multi-arm, multi-stage, adaptive, platform, phase III randomised, double-blind, placebo-controlled trial of repurposed drugs in motor neuron disease.

Journal:	*BMJ Open*
Manuscript ID	bmjopen-2022-064173
Article Type:	Protocol
Date Submitted by the Author:	25-Apr-2022

Complete List of Authors:	Wong, Charis; The University of Edinburgh Centre for Clinical Brain Sciences, Anne Rowling Regenerative Neurology Clinic; The University of Edinburgh Centre for Clinical Brain Sciences, Euan MacDonald Centre for Motor Neuron Disease Research<br>Dakin, Rachel; The University of Edinburgh Centre for Clinical Brain Sciences; The University of Edinburgh Centre for Clinical Brain Sciences, Anne Rowling Regenerative Neurology Clinic<br>Williamson, Jill; The University of Edinburgh Centre for Clinical Brain Sciences, Anne Rowling Regenerative Neurology Clinic; The University of Edinburgh Centre for Clinical Brain Sciences, Euan MacDonald Centre for Motor Neuron Disease Research<br>Newton, Judith; The University of Edinburgh Centre for Clinical Brain Sciences, Anne Rowling Regenerative Neurology Clinic; The University of Edinburgh Centre for Clinical Brain Sciences, Euan MacDonald Centre for Motor Neuron Disease Research<br>Steven, Michelle; The University of Edinburgh Usher Institute of Population Health Sciences and Informatics, Edinburgh Clinical Trials Unit<br>Colville, Shuna; The University of Edinburgh Centre for Clinical Brain Sciences, Anne Rowling Regenerative Neurology Clinic; The University of Edinburgh Centre for Clinical Brain Sciences, Euan MacDonald Centre for Motor Neuron Disease Research<br>Chataway, Jeremy; University College London Institute of Neurology Queen Square Multiple Sclerosis Centre, Neuroinflammation; National Institute for Health Research, University College London Hospitals<br>Swingler, Robert; The University of Edinburgh Centre for Clinical Brain Sciences, Euan MacDonald Centre for Motor Neuron Disease Research; London North West University Healthcare NHS Trust, Northwick Park Hospital<br>Parker, Richard; The University of Edinburgh Usher Institute of Population Health Sciences and Informatics, Edinburgh Clinical Trials Unit<br>Weir, Christopher; The University of Edinburgh Usher Institute of Population Health Sciences and Informatics, Edinburgh Clinical Trials Unit<br>Stallard, Nigel; University of Warwick, Statistics and Epidemiology, Division of Health Sciences, Warwick Medical School<br>Parmar, Mahesh; Medical Research Council Clinical Trials Unit at University College London, Institute of Clinical Trials and Methodology<br>Macleod, Malcolm; The University of Edinburgh Centre for Clinical Brain

	Sciences<br>Pal, Suvankar; The University of Edinburgh Centre for Clinical Brain Sciences, Anne Rowling Regenerative Neurology Clinic; The University of Edinburgh Centre for Clinical Brain Sciences, Euan MacDonald Centre for Motor Neuron Disease Research<br>Chandran, Siddharthan ; The University of Edinburgh Centre for Clinical Brain Sciences, Euan MacDonald Centre for Motor Neuron Disease Research; UK Dementia Research Institute, University of Edinburgh
Keywords:	Motor neurone disease < NEUROLOGY, Clinical trials < THERAPEUTICS, Neurology < INTERNAL MEDICINE, STATISTICS & RESEARCH METHODS

SCHOLARONE™
Manuscripts

TITLE

Motor Neuron Disease Systematic Multi-Arm Adaptive Randomised Trial (MND-SMART): A multi-arm, multi-stage, adaptive, platform, phase III randomised, double-blind, placebo-controlled trial of repurposed drugs in motor neuron disease.

AUTHORS

Charis Wong[1,2,3]*, Rachel S Dakin[1,2,3]*, Jill Williamson[1,2,3], Judith Newton[1,2,3], Michelle Steven[4], Shuna Colville[1,2,3], Jeremy Chataway[5,6,7], Robert J Swingler[2,8], Richard A Parker[4], Christopher J Weir[4], Nigel Stallard[9], Mahesh KB Parmar[7], Malcolm R Macleod[1], Suvankar Pal[ψ1,2,3], Siddharthan Chandran[ψ1,2,3,10].

**Joint first authors*

[ψ]Co-Corresponding authors:

Siddharthan Chandran PhD

Address: Centre for Clinical Brain Sciences, University of Edinburgh, Edinburgh, EH16 4SB, UK.

Email: Siddharthan.chandran@ed.ac.uk

Suvankar Pal

Address: Centre for Clinical Brain Sciences, University of Edinburgh, Edinburgh, EH16 4SB, UK.

Email: suvankar.pal@ed.ac.uk

Affiliations:

1. Centre of Clinical Brain Sciences, University of Edinburgh, Edinburgh, EH16 4SB, UK.
2. Euan Macdonald Centre for Motor Neuron Disease Research, University of Edinburgh, Edinburgh, EH16 4SB, UK.
3. Anne Rowling Regenerative Neurology Clinic, University of Edinburgh, Edinburgh, EH16 4SB, UK.
4. Edinburgh Clinical Trials Unit, Usher Institute of Population Health Sciences and Informatics, University of Edinburgh, Edinburgh, UK.
5. Queen Square Multiple Sclerosis Centre, Department of Neuroinflammation, UCL Queen Square Institute of Neurology, Faculty of Brain Sciences, University College London, London, United Kingdom WC1B 5EH
6. National Institute for Health Research, University College London Hospitals, Biomedical Research Centre, London, United Kingdom W1T 7DN
7. Medical Research Council Clinical Trials Unit at UCL, Institute of Clinical Trials and Methodology, University College London, London, United Kingdom WC1V 6LJ
8. London North West University Healthcare NHS Trust, Northwick Park Hospital, A404 Watford Rd, Harrow HA1 3UJ
9. Statistics and Epidemiology, Division of Health Sciences, Warwick Medical School, University of Warwick, Coventry, UK.
10. UK Dementia Research Institute Edinburgh, University of Edinburgh, Edinburgh, EH16 4SB, UK.

Word count: 3964 words

For peer review only

ORCID iD

Charis Wong 0000-0002-8488-037X

Rachel Dakin 0000-0002-3697-851

Jill Williamson 0000-0002-2263-5950

Jeremy Chataway 0000-0001-7286-6901

Richard Parker 0000-0002-2658-5022

Nigel Stallard 0000-0001-7781-1512

Mahesh Parmar 0000-0003-0166-1700

Malcolm R. Macleod 0000-0001-9187-9839

Suvankar Pal 0000-0003-4276-639X

Siddharthan Chandran 0000-0001-6827-1593

For peer review only

ABSTRACT

Introduction:

Motor neuron disease (MND) is a rapidly fatal neurodegenerative disease. Despite decades of research and clinical trials there remains no cure and only one globally approved drug, riluzole, which prolongs survival by 2-3 months. Recent improved mechanistic understanding of MND heralds a new translational era with many potential targe]ts being identified that are ripe for clinical trials. Motor Neuron Disease Systematic Multi-Arm Adaptive Randomised Trial (MND-SMART) aims to evaluate the efficacy of drugs efficiently and definitively in a multi-arm, multi-stage, adaptive trial. The first two drugs selected for evaluation in MND-SMART are trazodone and memantine.

Methods and analysis:

Initially, up to 531 participants (177/arm) will be randomised 1:1:1 to oral liquid trazodone, memantine and placebo. The co-primary outcome measures are the Amyotrophic Lateral Sclerosis Functional Rating Scale Revised (ALSFRS-R) and survival. Comparisons will be conducted in four stages. The decision to continue randomising to arms after each stage will be made by the Trial Steering Committee who receive recommendations from the Independent Data Monitoring Committee. The primary analysis of ALSFRS-R will be conducted when 150 participants/arm, excluding long survivors, have completed 18 months of treatment; if positive the survival effect will be inferentially analysed when 113 deaths have been observed in the placebo group. The trial design ensures that other promising drugs can be added for evaluation in planned trial adaptations. Using this novel trial design reduces time, cost, and number of participants required to definitively (phase III) evaluate drugs and reduces exposure of participants to potentially ineffective treatments.

Ethics and dissemination

MND-SMART was approved by the West of Scotland Research Ethics Committee on 2 October 2019. (REC reference: 19/WS/0123) Results of the study will be submitted for publication in a peer-reviewed journal and a summary provided to participants.

Trial registration numbers

EudraCT Number: 2019-000099-41. ClinicalTrials.gov Identifier: NCT04302870.

Keywords: motor neuron disease, amyotrophic lateral sclerosis, randomised controlled trial, adaptive trials, multi-arm multi-stage trials.

For peer review only

STRENGTHS AND LIMITATIONS OF THIS STUDY

- The Motor Neuron Disease – Systematic Multi-Arm Adaptive Randomised Trial (MND-SMART) is the first multi-arm multi-stage (MAMS) phase III trial in motor neuron disease to launch in Europe.
- This novel trial design aims to achieve efficiencies in time, cost, and requirements for sample size through planned trial adaptations: ineffective arms can be dropped at interim analysis whilst new arms can be added.
- Broad inclusion criteria allow wider participation and ensures generalisability of results in a heterogenous condition.
- Drugs are selected using a combined approach of a data-driven framework incorporating published literature and expert opinion.
- The complexities of the conduct of this MAMS trial necessitate particular operational considerations.

For peer review only

INTRODUCTION

Motor neuron disease (MND) is an incurable, rapidly progressive and invariably fatal disease characterised by selective motor neuron loss. Despite 125 phase II to III drug trials between 2008 and 2019, riluzole, licensed in the UK by the National Institute for Health and Care Excellence (NICE) in 2001, remains the only disease-modifying drug with marketing authorisation in the UK and Europe, with a median survival benefit of 2-3 months.[1 ,2] Across the UK historical MND trial participation is less than 5%.[2] Recently, edaravone and masitinib have emerged as promising candidates following positive trials in enriched trial populations.[3 ,4] Edaravone was licensed in Japan and USA in 2015 and 2017 respectively. However, evidence of generalisable survival benefit for these drugs remain limited; in a long-term prospective cohort study, edaravone did not improve survival or slow functional decline.[5]

There are many challenges facing clinical trials for MND ranging from comparatively blunt outcome measures to disease factors including rapid progression and heterogeneity. Benchmark outcomes for MND trials are largely ordinal clinical rating scales and survival [2 ,6]. To detect a significant treatment effect using these measures, large sample sizes are often needed to achieve adequate study power, especially when using traditional fixed two-arm designs. To account for heterogeneity, many previous trials used strict inclusion criteria.[2 ,7] However, a mean exclusion rate of 60% in 38 previous trials did not reduce heterogeneity in survival or functional decline.[7] Strict inclusion criteria, diagnostic delay and the rapidly progressive and fatal nature of MND often results in a short time window for people with MND (pwMND) to participate in trials. Furthermore, trial retention is often poor – in a recent review, 40% of trials reported attrition rates ≥20%.[2] Adherence with trial protocol often becomes challenging with increasing disability. Movement, speech, swallowing and respiration are commonly affected, the latter causing some pwMND to be dependent on enteral feeding and ventilatory support. Cognitive and behavioural changes also occur in approximately 35% of pwMND with up to 15% having frontotemporal dementia (FTD).[8 ,9] Against this background, there have been major advances in our understanding of the pathobiology of MND as well as an increasing number of candidate medicines being identified supporting innovation in trial design for MND.

Multi-arm multi-stage (MAMS) and other adaptive platform trials have been used successfully in other fields, especially cancer medicine and infectious diseases, and are particularly effective where there are several promising medicines without clear *a priori* evidence to favour one over the other.[2 ,10 ,11] A multi-arm trial allows simultaneous evaluation of multiple treatments against a single control group. An adaptive trial design allows additional arms to be added in what is in effect a "continuous" trial platform. These features can provide major efficiency gains in time, cost and sample size requirements compared to running multiple (and almost certainly consecutive)

conventional two-arm studies, each with their own control arm. Beside MND-SMART trial, there are two other declared adaptive platform trials in MND: USA-based HEALEY amyotrophic lateral sclerosis Platform Trial[12] (ClinicalTrials.gov registration number NCT04297683) and European-based Treatment and Research Initiative to Cure amyotrophic lateral sclerosis 'MAGNET' platform trial.[13]

Drug repurposing has proven successful in many areas, including multiple sclerosis.[14] The lower development cost and shorter development timelines with availability of long-term safety data makes this a particularly attractive option for devastating conditions like MND where trial attrition rates are historically high.[2] Rapid advances in genomics, molecular and human stem cell technology has led to better understanding of the underlying pathobiology of MND and discovery of novel drug targets. Computational advances have made living systematic reviews of the entire published literature of MND and other neurodegenerative diseases which may share similar pathways feasible, thus providing a robust, comprehensive and continually updated evidence-base to identify, evaluate and prioritise candidate drugs for trials.[15-17] These principles were used to inform drug selection for the Multiple Sclerosis-Secondary Progressive Multi-Arm Randomisation Trial (MS-SMART), a multi-arm trial of repurposed drugs in progressive multiple sclerosis.[18 ,19] The initial candidate drugs for MS-SMART based on this approach were ibudilast, amiloride and riluzole, but due to drug supply issue, ibudilast was switched to fluoxetine. Although the drugs tested in MS-SMART did not show clear evidence of activity, ibudilast slowed progression of brain atrophy in a separate phase II trial.[20]

Against this background, Motor Neuron Disease – Systematic Multi-Arm Randomised Trial (MND-SMART) is a phase III randomised MAMS trial of repurposed drugs in MND. The first two candidate drugs to be tested are trazodone and memantine.

METHODS AND ANALYSIS

Trial objectives

The primary objective of MND-SMART is to assess the clinical effects of putative neuroprotective drugs compared to placebo as measured by rate of change in score of the Amyotrophic Lateral Sclerosis Functional Rating Scale Revised (ALSFRS-R), and conditional on identifying benefit on this, to assess the effect of these drugs on survival. Secondary objectives are to assess the safety profile of candidate drugs, and their effects on time to King's stage 4a (nutritional failure), time to King's stage 4b (respiratory failure), cognitive function and behaviour as assessed using the Edinburgh Cognitive and Behavioural ALS Screen (ECAS), respiratory function measured by forced

For peer review only

vital capacity (FVC), anxiety and depression measured by the Hospital Anxiety and Depression Scale (HADS), and quality of life evaluation using EQ-5D-5L.

Overview of design

MND-SMART is a phase III double-blind, randomised, placebo-controlled MAMS platform trial. MND-SMART will initially assess the efficacy of memantine (Arm A) and trazodone (Arm B) against a single contemporaneous placebo control arm (Arm C) with investigational medicinal products (IMPs) delivered as liquids. Participants are randomised 1:1:1 to Arms A, B and C with up to 531 pwMND (177/arm) randomised to complete the analysis of these arms (Figure 1). Allowing for early withdrawal and long survivors, this will give 150 participants/group in the primary analysis of ALSFRS-R. Patients will have five appointments in the first four to eight weeks followed by assessments every two months, most completed remotely, and two appointments for treatment cessation when this is required (Figure 2).

The ALSFRS-R comparisons will be conducted in three stages (Figure 1) with the opportunity to stop randomisation to arms that do not meet the predefined continuing criteria (Table 1) at the end of stages 1 of 2. Stage 1 will be completed when 50 participants/arm (excluding long survivors defined as >8 years since diagnosis at baseline) have completed 6 months of treatment and Stage 2 will be completed when 100 participants per arm (excluding long survivors) have completed 12 months of treatment. The primary analysis (stage 3) will be conducted based on rate of decline of ALSFRS-R when 150 participants/arm (excluding long survivors) complete 18 months of treatment. Participants will continue to take their allocated treatment until 113 deaths have been observed in the placebo group, or stage 3 analysis data is collected – whichever is later, with the primary analysis of survival being performed at this point.

For peer review only

Table 1 Timing and guidance for Stage 1 and Stage 2 analyses

**Stage**	**Number of pwALS/arm excluding long survivors**	**Criterion to continue to next stage**
Stage 1	50 with ≥6 months follow-up	95%CI of the rate of change in ALSFRS-R compared to placebo must include a relative improvement of 25% in the rate of decline
Stage 2	100 with ≥12 months follow-up	Improvement in rate of change in ALSFRS-R compared to placebo significant at pairwise one-sided 10% level

The study protocol follows the Standard Protocol Items: Recommendations for Interventional Trials guidelines[21] and was registered on the European Clinical Trials Registry (2019-000099-41) on 25th July 2019 and ClinicalTrials.gov (NCT04302870) on 10th March 2020.

Planned trial adaptations

In addition to stopping arms that do not meet continuing criteria at the end of stage 1 and 2, we intend to introduce new arms in the future by substantial amendment. Any new arm will be compared to contemporaneous controls with each comparison following the multi-stage process described above. As long as treatments are distinct, for example belonging to different classes of medicine, no multiplicity adjustments will be made in analysis of pairwise comparisons.[22-24]

Re-entry of participants into the trial

Participants will be able to enter the trial on more than once occasion, under certain conditions. To ensure a participant does not contribute data to more than one arm in the same treatment comparison, they will only be able to re-enter MND-SMART if they satisfy one of the following criteria: the arm they were randomised to has been dropped; they are in the control group and all experimental arms open at the time at which they were randomised have been dropped; or their data has been included in a final survival-based comparison. Any participant re-entering the trial will be screened for eligibility in the same way as a trial naïve individual. Previous trial entry will be noted to ensure their medical

For peer review only

and treatment history is linked to allow for long-term follow up of potential signals and understanding of investigational drugs exposed to.

Participants, interventions and outcomes

Trial setting

MND-SMART is recruiting across the UK with 17 recruiting centres (listed on mnd-smart.org) at 10th March 2022. Research staff will enter data on site via a secure electronic case report form (eCRF), with quality control and analysis conducted independently by Edinburgh Clinical Trials Unit (ECTU).

Eligibility criteria

Eligibility criteria for MND-SMART are shown in Table 2.

For peer review only

Table 2 MND-SMART eligibility criteria (protocol V 6.0). MND, Motor neuron disease; ALT, Alanine aminotransferase; ALP, alkaline phosphatase; GGT, gamma-glutamyltransferase; eGFR, estimated glomerular filtration rate; TSH, thyroid-stimulating hormone.

**Inclusion criteria**	
• Confirmed diagnosis of MND (including the following subtypes: Amyotrophic Lateral Sclerosis by El Escorial Criteria (possible, probable, and definite), Primary Lateral Sclerosis, and Progressive Muscular Atrophy).<br>• Over 18 years of age.<br>• Women of childbearing potential must have a negative pregnancy test within 7 days prior to, or at, the baseline visit.	• Women of childbearing potential and fertile men must be using an appropriate method of contraception to avoid any unlikely teratogenic effects of the selected drugs from time of consent, to 4 weeks after treatment inclusive.<br>• Willing and able to comply with the trial protocol and ability to understand and complete questionnaires.<br>• Written informed consent (this can be signed by a proxy in the case of limb dysfunction).
**Exclusion criteria**	
• Diagnosis of Frontotemporal Dementia (FTD-MND) or any other significant psychiatric disorder that prevents informed consent being given.<br>• Patients in the manic phase of bipolar disorder.<br>• Alcoholism (self-reported)<br>• Active suicide ideation assessed using the Columbia-Suicide Severity Rating Scale.<br>• On concurrent investigational medication (including biological therapy).<br>• Known hypersensitivity, including hereditary fructose intolerance, or adverse reaction to the active substances and their excipients or any past medical history contraindicating use of any of the investigational medicinal products (IMPs).<br>• Pregnancy or breast-feeding females.<br>• If ALT, ALP, bilirubin or GGT >3 times the upper limit of normal; creatinine clearance (creatinine clearance or eGFR) <30 ml/min; or serum free T4 >25pmol/l or TSH <0.2mU/l.	• If corrected QT interval on 12 lead ECG >450 ms.<br>• Diagnosis of ventricular arrhythmias, heart block or in the immediate recovery period after myocardial infarction (< 6 weeks).<br>• Already taking any of the IMPs in the protocol.<br>• Contraindications to any of the IMPs according to Summary of Product Characteristics (SPC).<br>• Taking a medication that interacts with the active substances and their excipients according to the SPCs, including but not limited to; Dextromethorphan, Amantadine; Ketamine, Monoamine-oxidase inhibitors ((MAOIs): Rasagiline, Selegiline, Safinamide, Tranylcypromine, Phenelzine, Isocarboxazid, Moclobemide).<br>• Patients who the PI considers will not be able to comply with the study protocol

For peer review only

Ascertainment and recruitment

Potential participants are identified through self-referral from potential participants who may be made aware of the trial by the media, literature or website (www.mnd-smart.org), by members of clinical and care teams, and using the existing National MND Care/Research platform (CARE-MND) in Scotland and other UK wide MND registries that contain contact details of people who have consented to be contacted directly about relevant research opportunities. Potential participants will be informed about the study and given a patient information sheet (PIS). They can be pre-screened by the individual's clinical team or, in Scotland, against trial criteria using information from CARE-MND.

The screening visit includes documentation of written informed consent. If a participant is physically unable to give written consent due to limb dysfunction, verbal consent can be given with a witness independent of the trial signing the consent form. A clinical trial physician will evaluate each potential participant against the trial eligibility criteria and recruit participants. Each centre is required to maintain an anonymised log of all ineligible patients and eligible patients who declined participation.

The first participant was randomised on 27th February 2020.

Identifying the research interventions for priority evaluation in MND-SMART

For peer review only

We conducted a two-stage systematic review of the clinical literature of MND and four other neurodegenerative diseases (Alzheimer's disease, Parkinson's disease, Huntington's disease, and Multiple Sclerosis) which may share similar pathways, and of the preclinical MND literature to inform identification and selection of investigational drugs for MND-SMART by an expert panel.[25]Through this structured process memantine and trazodone were selected for evaluation. Memantine is a non-competitive N-methyl-D-aspartate (NMDA) receptor antagonist used in the management of Alzheimer's disease. It was shown to significantly delay disease progression and improve survival in mouse models carrying a high copy number of $SOD1^{G93A}$.[26] Three early phase MND clinical trials using memantine showed some evidence of safety and potential signals for efficacy in ALSFRS-R and in cerebrospinal and neurophysiological biomarkers.[27-29] Trazodone is an atypical antidepressant drug of the serotonin antagonist and reuptake inhibitor class. In an unbiased drug screen, trazodone inhibited Protein Kinase RNA-like endoplasmic reticulum kinase (PERK), which is pivotal to stress granule formation, a common feature of neurodegenerative diseases.[30] Inhibition of PERK was found to be beneficial in an ALS fly model as well as in an *in vitro* neuronal assay of TDP-43 injury.[31] Modulation of ER-stress response by trazodone resulted in improvement in survival of prion disease and Frontotemporal dementia (FTD) animal models.[30] In Huntington's disease mouse models, trazodone improved behavioural outcomes and exerted mitochondrial protective effects,[32] a pathway of interest in MND.[33] In a phase II trial in 31 people with FTD, trazodone improved cognition as assessed by the neuropsychiatric inventory.[34]

Drug allocation, titration and monitoring

Participants are randomised to memantine hydrochloride 10 mg/5 ml oral solution, trazodone hydrochloride 100 mg/5 ml oral solution or placebo oral solution containing no active pharmaceutical ingredient via a web-based randomisation system to maintain allocation concealment. Drugs and placebo are manufactured by Huddersfield Pharmacy Specials as liquids with identical colour, flavour and consistency. Weekly, during the first 4 weeks of the study, a clinical trial physician assesses participants for adverse events (AEs) and tolerability. IMP dose is also titrated using the dosing schedule shown in Table 3. Participants are asked to record adherence, drug doses and any reasons for non-adherence.

For peer review only

Table 3 MND-SMART dose titration schedule

	Baseline (Week 0)<br>'quarter dose':<br>2.5 ml	Week 1<br>'half dose':<br>5 ml	Week 2<br>'three quarter dose':<br>7.5 ml	Week 3<br>'full dose':<br>10 ml
Memantine	5 mg	10 mg	15 mg	20 mg
Trazodone	50 mg	100 mg	150 mg	200 mg
Placebo	2.5 ml	5 ml	7.5 ml	10 ml

Participants may stop treatment early with appropriate down-titration due to unacceptable AEs, pregnancy, inter-current illness that prevents further treatment, and any changes in the participant's condition justifying discontinuation of treatment according to investigators' discretion, including as part of end-of-life care.

Concomitant care and interventions

All concomitant medications are permitted except dextromethorphan, amantadine, ketamine, rasagiline, selegiline, safinamide, tranylcypromine, phenelzine, isocarboxazid and moclobemide. Participants are allowed to start standard of care, including riluzole and interventions like gastrostomy and non-invasive ventilation (NIV), after randomisation.

Outcome measures

Primary outcome measures

ALSFRS-R and survival are co-primary outcomes in MND-SMART. ALSFRS-R assesses MND-related disability in gross and fine motor tasks, bulbar function and respiratory status.[35] This scale has been widely used and validated as an appropriate and accurate assessment of functional ability and disease progression. It is easy to administer and has been validated for administration in person or over the telephone or by videoconferencing with high inter-rater and intra-rater reliability.[36]

Secondary outcomes measures

Secondary outcomes for MND-SMART include time to King's stage 4a and time to King's stage 4b, cognitive function and behaviour as assessed using ECAS, FVC, HADS and EQ-5D-5L quality of life evaluation.

Exploratory outcome

For peer review only

An exploratory outcome investigating ALSFRS-R scores post 18 months participation is also included.

Safety and tolerability outcomes

All study visits will capture data on AEs and adherence.

Participant timeline

Participant timeline and visit schedule is shown in Figure 2 and Table 4. Most study visits can be conducted by telephone or secure videoconferencing.

Table 4 MND-SMART visit and assessment schedule. [A]These appointments can be completed using video conferencing or telephone call. [B] These appointments should be completed face to face wherever possible. * For women of childbearing potential. † If more than 7 days from previous test. ¥ modified King's staging will take place at remote appointments ✓ start IMP. ‡ this assessment includes an interview with a partner/relative/friend/carer if the participant has consented. If an arm is dropped the IMP dose should be halved at the next scheduled appointment and participants then complete the two treatment cessation appointments. Appointments from 15 onwards are the same to be repeated every 2 months

For peer review only

Appointment	Time	Informed consent	Inclusion/exclusion review	Eligibility confirmation	Demography	Disease history	Past medical history	Current medications	Safety bloods	ECG	Suicide ideation (CSSRS)	Pregnancy test	Randomisation	FVC	ALSFRS-R & King's staging¥	EQ-5D-5L	HADS	ECAS ‡	Sub-studies	Research bloods	Drug supply	Titration up	Compliance check	Adverse Events
**1 Screening**		x	x	x	x	x	x	x	x	x	x	x*							x					x
**2 Baseline**	**Time 0**		x	x				x				x†	x	x	x	x	x	x	x	x	x	✓		x
**3, 4, 5 [A]**	**Weeks 1, 2 and 3**							x														x	x	x
**6, 7, 9, 10, 12, 13 [A]**	**Month 2, 4, 8, 10, 14, 16**							x							x				x		x		x	x
**8 [B]**	**Month 6**							x						x	x	x	x		x	x	x		x	x
**11 [B]**	**Month 12**							x						x	x	x	x	x	x	x	x		x	x
**14 [B]**	**Month 18**							x						x	x	x	x	x	x	x	x		x	x
**15, 16, 17… [A]**	**Month 20, 22, 24…**							x							x						x		x	x

For peer review only

Number of Patients required

For sample size calculations, simulation studies were performed using data from Pooled Resource Open-Access ALS Clinical Trials (PRO-ACT) Open Access ALS Clinical Trial database which pooled data from several MND clinical trials.[37 ,38] The simulations included interim analyses at the end of stages 1 and 2. For arms continuing beyond the second stage, the primary analysis was simulated based on 18-month follow-up data for 150 participants/arm excluding long survivors. This primary analysis plan is described in the Statistical Methods section below, and it should be noted that the design is calculated controlling the pairwise error rate at 2.5% (one-sided). To allow for a drop-out rate of approximately 10%, and for the exclusion from the analysis of long survivors (estimated to make up 5% of the trial population), we will randomise 177 participants to each treatment arm (total 531).

The simulations demonstrated that for a true 25% reduction in the rate of decline of ALSFRS-R, the sample size is sufficient to give a probability of 86% of continuing beyond the stage 2 analysis into the final stage and a probability of continuing to the final stage and obtaining a significant result of 83%, equivalent to an independent phase III trial power of 90%.The probability of dropping an active arm at the first interim analysis is 23% if the true treatment effect is identical to the control group and 2% if there is a true 25% reduction in the average decrease in ALSFRS-R in that arm.

With regards to the survival co-primary outcome, follow-up will continue until 113 deaths have been observed in the placebo arm. With 113 deaths/arm, this test will have 90% power to detect a hazard ratio of 0.65. To maintain overall type I error control at 2.5% (one-sided), survival will be analysed inferentially for an IMP only if statistically significant benefit has been demonstrated on ALSFRS-R. In the situation that the ALSFRS-R outcome measure does not produce a statistically significant result overall survival data will be analysed to provide supporting information but will not provide pivotal evidence of efficacy.

Assignment of interventions

Treatment allocation and blinding

Randomisation is performed at baseline visit via a secure web-based randomisation system employing the following minimisation variables: riluzole use, use of NIV and/or gastrostomy, and long survival (>8 years from diagnosis). The allocation sequence incorporates a random element based on computer-generated pseudo-random numbers to maintain unpredictability. Participants, investigators, care providers and outcome assessors are blinded to treatment allocation. To maintain blinding, each bottle is identified by a unique bottle number. In situations where medical management necessitates

For peer review only

knowledge of the study medication, emergency unblinding can be done through the web-based randomisation system.

Data Collection, management and analysis

Data collection and management

Data is collected by trained site staff and entered into the eCRF via a web-based portal. Access to the eCRF is role-based and delegated site staff are assigned unique usernames. Activity on the eCRF is tracked and auditable. The trial database includes in-built measures to reduce data entry errors and ensure data quality. Regular central data checks are performed with queries and follow up undertaken on a site by site basis. The eCRF is hosted by the University of Edinburgh in their secure environment as detailed in the trial data management plan.

Statistical methods

A CONSORT flow diagram will be reported. Descriptive statistics will be used to summarise baseline characteristics for each treatment group. Participants will be included in the analysis if they were not long survivors at baseline and have at least one ALSFRS-R score recorded either at baseline or during follow-up.

A hierarchical normal linear model will be used to compare the rate of decrease in ALSFRS-R over the follow-up period in each of the two active treatment group arms with the placebo arm. The analysis will adjust for the effects of baseline minimisation variables. Long survivors at baseline are not included in the primary ALSFRS-R analysis and so adjustment for this minimisation variable is not required. Estimates and 95% confidence intervals will be obtained for the difference in ALSFRS-R decrease for each active treatment group minus placebo. At the first interim analysis, each experimental arm will continue to the second stage if the upper limit of its 95% confidence interval for the rate of change of ALSFRS-R compared to placebo lies below a 25% reduction in the rate of decline. For example, a 95% confidence interval for reduction in rate of decline of (-10%, 30%) would lead to continuation of the experimental arm, whereas the interval (-20%, 15%) would result in discontinuation.

At the second interim analysis, if a significant treatment benefit relative to the placebo is observed at the pairwise two-sided 20% significance level, then the treatment will proceed to the final (end of stage 3) analysis. At the final analysis, each pairwise comparison of trazodone and memantine with placebo, will be tested using a one-sided 2.5% significance. Most types of intercurrent events will be handled in the estimand using the treatment policy strategy, except for deaths which will be handled

For peer review only - http://bmjopen.bmj.com/site/about/guidelines.xhtml

using the hypothetical strategy. A detailed statistical analysis plan will contain full information on the estimands for ALSFRS-R and survival.

Analysis of the survival co-primary outcome at the end of stage 4 will be based on all participants, including long survivors at baseline. Kaplan Meier statistics will estimate the survival function and the median survival will be calculated for each group with 95% confidence intervals. Log-rank tests will be used to compare treatment and placebo survival curves and treatment effects will be estimated by a Cox proportional hazard model including covariates to allow for the effects of the baseline minimisation variables.

Pre-planned subgroup analyses of primary and secondary outcome measures will be performed for: participants with different survival at baseline (>8 years, < 8 years); participants with different subtypes of MND (amyotrophic lateral sclerosis, primary lateral sclerosis and progressive muscular atrophy); participants with C9orf72 expansions; participants with bulbar-onset and limb-onset forms of MND; participants age <40 or age >80; participants receiving support with NIV at randomisation and participants receiving feeding support with gastrostomy at randomisation..

Missing data handling methods will be described in the full statistical analysis plan.

Monitoring

Data monitoring

An Independent Data Monitoring Committee (IDMC) oversees the safety of participants in the trial and review interim analyses. The committee regularly monitors safety data and makes recommendations on whether the trial should continue as planned and on the dropping of treatment arms following formal interim analyses. The committee is composed of independent members, without competing interests, and meetings are conducted in accordance with the IDMC charter.

Adverse Events (AEs)

Detection, recording, and reporting adverse events

Participants are asked about the occurrence of AEs at every study appointment. AE data is also available from participant diaries and laboratory results. Each AE will be assessed at site for seriousness, causality and severity. A central unblinded team assesses the expectedness of all adverse reactions and serious adverse reactions. AEs due to pre-existing medical conditions are only reported if medically judged to be an unexpected worsening during the study. AEs related to MND of a participant should not be recorded for the trial if consistent with disease progression.

For peer review only - http://bmjopen.bmj.com/site/about/guidelines.xhtml

Withdrawal of study participants and early termination of trial

Participants are free to withdraw from the study at any point or a participant can be withdrawn by investigators. If withdrawal occurs, the primary reason will be documented in the participant's case record form. Discontinuing study drug does not constitute withdrawal – all attempts should be made to follow up the participant as per protocol and/or to re-commence treatment (if discontinuation was due to AEs which have subsequently resolved). Participants can choose to stop all appointments but remain in the trial for follow-up of survival from medical records only. Withdrawn subjects will not be replaced. The IDMC can request trial arm suspension or termination, for example due to unacceptable AEs.

Auditing

University of Edinburgh and NHS Lothian Academic and Clinical Central Office for Research and Development (ACCORD) is the trial sponsor. ACCORD personnel, or designees, will perform monitoring activities and study audits in accordance with the trial monitoring plan. Data is continually audited by the central team for return rate and completeness.

Patient and public involvement

Patient public involvement (PPI) has been crucial throughout the development of MND-SMART. Through support groups and a PPI advisory group, pwMND, their families and carers were consulted and provided suggestions to optimise trial recruitment and retention. These included limiting number of appointments or travel to appointments, use of remote appointments supported by telehealth, delivery of IMP to participants' homes where necessary, use of liquid IMP formulations to enable participants with swallowing difficulties including those using gastrostomy to participate and remain in trial, and using broad inclusion criteria where the main exclusions are limited to contraindications to study drugs and lack of capacity to consent.

ETHICS AND DISSEMINATION

Research ethics approval

MND-SMART was approved by the West of Scotland Research Ethics Committee on 2 October 2019. (REC reference: 19/WS/0123).

Protocol amendments

Protocol versions and substantial amendments to date are summarised in Table 5.

For peer review only

Table 5 Protocol versions and substantial amendments to date.* protocol v2.0 was given CTA

Protocol update	Protocol version	Protocol date	Reason for amendment
NA	V1	11th July 2019	N/A
Substantial amendment	V2*	20th August 2019	Addition of Clinical Trials Coordination and Facilitation Group (CTFG) definition of women of childbearing potential; and information on highly effective contraceptive methods. Additional pregnancy test added at the end of systemic exposure for women of childbearing potential
Substantial amendment – SA01	V3	24th October 2019	Clarification on trial design:<br>- Treatment and follow up duration will continue until 200 patients/group have 18 months follow up (whichever is later)<br><br>Guidance on prioritising primary endpoints, completing modified King's staging in remote appointments and timescale for completing carer part of the ECAS, within 2 weeks of participant part of assessment.<br><br>Addition that adverse events that are consistent with pre-existing medical conditions and/or the progression of MND do not need to be recorded in the CRF or reported (if serious) but should be noted in medical records.<br><br>Confirmation of the procedure for assigning expectedness to adverse reactions by the central expectedness team.
Substantial amendment – SA02	V3	24th October 2019	Addition of site
Substantial amendment – SA03	V3	24th October 2019	Addition of sites
Substantial amendment – SA04	V3	24th October 2019	Change of PI
Substantial amendment – SA05	V5	1st March 2021	Updates to exclusion criteria<br>Confirmation on pre-screening process and addition of remote consent using electronic software.<br>Update to allow use of clinical results for eligibility review and outcome assessments

For peer review only - http://bmjopen.bmj.com/site/about/guidelines.xhtml

			and addition of possibility for CI to unblind. Confirmation patients who withdraw can consent to follow-up of survival from medical records. Addition of ability to cross cover for sites and updates to requirements to courier drugs direct to participants including removing need to return IMP bottles to site for compliance. Addition of sending patient reported outcome questionnaires direct by email and clarification on assessments which can be performed remotely.
Substantial amendment – SA06	V5	1st March 2021	Addition of sites
Substantial amendment – SA07	V6	1st November 2021	Update to sample size based on move from family wise to pair wise Type I error control. Removal of SNIP (sniff nasal inspiratory pressure) as a secondary outcome measure. Confirmation removal of IMPs from trial does not constitute study end, new IMPs will be added. Addition of exploratory objective to investigate ALS_FRS-R post 18months participation. Clarifications on consent for patients with limb dysfunction, co-enrolment, IMP discontinuation and other points.

Confidentiality and access to data

All laboratory specimens, evaluation forms, reports, and other records are identified with as little personal information as possible to maintain participant confidentiality. Access to personal information is restricted to individuals from the research team treating the participants, representatives of the Sponsor(s) and regulatory authorities. Those analysing samples or data will not have access to personal information. The trial master file is held by the Anne Rowling Clinic and the database is held by ECTU for a minimum of 25 years from the defined end of study point.

Dissemination policy

To ensure data transparency, the results of any closed comparisons will be published in a peer reviewed journal as soon as it is possible when the integrity of the trial will not be affected.[39] Result of all analyses will be published. To maintain the scientific integrity of the study, data will not be released prior to the first publication of the results of the primary endpoint analysis, without the permission of the Trial Steering Committee (TSC). The TSC will agree a publication plan and must be consulted prior to release or publication of any study data. A summary of results will be provided to all participants via newsletters and the trial website.

For peer review only

ACKNOWLEDGMENTS

The authors would like to thank all the MND-SMART participants and collaborators involved in the trial. Particular thanks to the trial public patient involvement group who informed trial design and patient facing documents. The authors would like to thank the members of the Independent Data Monitoring Committee: Stephen Wroe (Chair), Camille Caroll, Sue Todd; and the members of the Trial Steering Committee: Helen Ford (Chair), Hadi Manji, James Carpenter, Beverley Gray, and former member Alan Gray. Additional thanks to Ronnie Harkess for designing the trial database and Catriona Keerie for statistical analysis. Particularly thanks also to all research nurses and practitioners involved in the trial.

MND-SMART is co-sponsored by University of Edinburgh and NHS Lothian Academic and Clinical Central Office for Research and Development (ACCORD).

The current MND-SMART sites and investigators are; Aberdeen Royal Cornhill Hospital, NHS Grampian, Dr Callum Duncan; Barts Health NHS Trust, Royal London Hospital, Dr Aleksandar Radunovic; Birmingham Queen Elizabeth Hospital, University Hospitals Birmingham NHS Foundation Trust, Dr Venkataramanan Srinivasan; Cambridge University Hospitals NHS Foundation Trust, Dr Rhys Roberts; Cardiff and Value University Health Board, University Hospital Wales, Dr Ken Dawson; Dundee Ninewells Hospital, NHS Tayside, Dr Ian Morrison, Edinburgh Anne Rowling Regenerative Neurology Clinic, NHS Lothian, Dr Suvankar Pal, Royal Devon and Exeter NHS Foundation Trust, Dr Timothy Harrower, Glasgow Queen Elizabeth University Hospital, NHS Greater Glasgow and Clyde, Dr George Gorrie, Inverness Raigmore Hospital, NHS Highland, Dr Javier Carod-Artal, Ipswich Hospital, East Suffolk and North Essex NHS Foundation Trust, Dr Clare Galton, Newcastle MND Care and Research Centre, Newcastle upon Tyne Hospitals NHS Foundation Trust, Dr Timothy Williams; Norfolk and Norwich University Hospitals NHS Foundation Trust, Dr Godwin Mamutse; Salford Royal NHS Foundation Trust, Dr Hisham Hamdalla, University Hospital Southampton NHS Foundation Trust, Dr Ashwin Pinto, St George's University Hospital NHS Foundation Trust, Dr Pablo Garcia Reitboeck; West Suffolk NHS Foundation Trust, Dr Francesca Crawley.

AUTHORS' CONTRIBUTIONS:

SCh is the chief investigator and provides clinical expertise and with SP and MKBP is responsible for study concept and design. CW, JN, JC and RJS provide clinical and scientific expertise and helped with design of the study. RAP, CJW, NS and MKBP provided statistical expertise for trial design. RAP and CJW are the trial statisticians and provide expertise in day to day running of the trial. RSD, JW and SCo contributed to study design and trial management. MS is the data manager and provided expertise for trial design and day to day management. RSD is the trial manager and responsible for the running of the trial. CW and RSD contributed equally to this paper.

For peer review only

FUNDING STATEMENT

MND-SMART is funded by grants from MND Scotland, My Name'5 Doddie Foundation (DOD/14/15) and specific donations to the Euan MacDonald Centre. CJW, RAP and MS were supported in this work by NHS Lothian via Edinburgh Clinical Trials Unit.

The study funders have no role in study design, data collection, data analysis, data interpretation, or writing of publications.

For the purpose of open access, the author has applied a Creative Commons Attribution (CC BY) licence to any Author Accepted Manuscript version arising from this submission.

COMPETING INTERESTS STATEMENT

CJW is a member of the independent data monitoring committee for a trial of masitinib in ALS, for which his institution receives funding from AB Science.

In the last 3 years, JC has received support from the Efficacy and Evaluation (EME) Programme, a Medical Research Council (MRC) and National Institute for Health Research (NIHR) partnership and the Health Technology Assessment (HTA) Programme (NIHR), the UK MS Society, the US National MS Society and the Rosetrees Trust. He is supported in part by the NIHR University College London Hospitals (UCLH) Biomedical Research Centre, London, UK. He has been a local principal investigator for a trial in MS funded by the Canadian MS society. A local principal investigator for commercial trials funded by: Actelion, Novartis and Roche; and has taken part in advisory boards/consultancy for Azadyne, Janssen, Merck, NervGen, Novartis and Roche.

For peer review only

REFERENCES

1. Miller RG, Mitchell JD, Lyon M, et al. Riluzole for amyotrophic lateral sclerosis (ALS)/motor neuron disease (MND). *Cochrane Database Syst Rev* 2002(2):CD001447.
2. Wong C, Stavrou M, Elliott E, et al. Clinical trials in amyotrophic lateral sclerosis: a systematic review and perspective. *Brain Commun* 2021;**3**(4):fcab242.
3. Mora JS, Genge A, Chio A, et al. Masitinib as an add-on therapy to riluzole in patients with amyotrophic lateral sclerosis: a randomized clinical trial. *Amyotroph Lateral Scler Frontotemporal Degener* 2020;**21**(1-2):5-14.
4. Safety and efficacy of edaravone in well defined patients with amyotrophic lateral sclerosis: a randomised, double-blind, placebo-controlled trial. *Lancet Neurol* 2017;**16**(7):505-12.
5. Witzel S, Maier A, Steinbach R, et al. Safety and Effectiveness of Long-term Intravenous Administration of Edaravone for Treatment of Patients With Amyotrophic Lateral Sclerosis. *JAMA Neurol* 2022.
6. van den Berg LH, Sorenson E, Gronseth G, et al. Revised Airlie House consensus guidelines for design and implementation of ALS clinical trials. *Neurology* 2019;**92**(14):e1610-e23.
7. van Eijk RPA, Westeneng HJ, Nikolakopoulos S, et al. Refining eligibility criteria for amyotrophic lateral sclerosis clinical trials. *Neurology* 2019;**92**(5):e451-60.
8. Crockford C, Newton J, Lonergan K, et al. ALS-specific cognitive and behavior changes associated with advancing disease stage in ALS. *Neurology* 2018;**91**(15):e1370.
9. Phukan J, Elamin M, Bede P, et al. The syndrome of cognitive impairment in amyotrophic lateral sclerosis: a population-based study. *Journal of Neurology, Neurosurgery & Psychiatry* 2012;**83**(1):102.
10. Parmar MK, Sydes MR, Cafferty FH, et al. Testing many treatments within a single protocol over 10 years at MRC Clinical Trials Unit at UCL: Multi-arm, multi-stage platform, umbrella and basket protocols. *Clin Trials* 2017;**14**(5):451-61.
11. Mehta AR, Chataway J, Pal S, et al. Trials for neurodegenerative diseases: time to innovate. *Lancet Neurol* 2021;**20**(12):984.
12. Paganoni S, Berry JD, Quintana M, et al. Adaptive Platform Trials to Transform Amyotrophic Lateral Sclerosis Therapy Development. *Ann Neurol* 2022;**91**(2):165-75.
13. van Eijk RPA, Kliest T, McDermott CJ, et al. TRICALS: creating a highway toward a cure. *Amyotroph Lateral Scler Frontotemporal Degener* 2020;**21**(7-8):496-501.
14. Pushpakom S, Iorio F, Eyers PA, et al. Drug repurposing: progress, challenges and recommendations. *Nat Rev Drug Discov* 2019;**18**(1):41-58.
15. Bannach-Brown A, Przybyla P, Thomas J, et al. Machine learning algorithms for systematic review: reducing workload in a preclinical review of animal studies and reducing human screening error. *Systematic reviews* 2019;**8**(1):23.
16. Elliott JH, Synnot A, Turner T, et al. Living systematic review: 1. Introduction-the why, what, when, and how. *Journal of clinical epidemiology* 2017;**91**:23-30.
17. Wong C, Liao J, Gregory J, et al. Study protocol: ReLiSyR-MND (Repurposing Living Systematic Review – Motor Neuron Disease): A systematic approach to identify neuroprotective interventions for motor neuron disease Secondary Study protocol: ReLiSyR-MND (Repurposing Living Systematic Review – Motor Neuron Disease): A systematic approach to identify neuroprotective interventions for motor neuron disease 2021. https://osf.io/5jp43.
18. Connick P, De Angelis F, Parker RA, et al. Multiple Sclerosis-Secondary Progressive Multi-Arm Randomisation Trial (MS-SMART): a multiarm phase IIb randomised, double-blind, placebo-controlled clinical trial comparing the efficacy of three neuroprotective drugs in secondary progressive multiple sclerosis. *BMJ open* 2018;**8**(8):e021944.
19. Vesterinen HM, Connick P, Irvine CM, et al. Drug repurposing: a systematic approach to evaluate candidate oral neuroprotective interventions for secondary progressive multiple sclerosis. *PloS one* 2015;**10**(4):e0117705.
20. Fox RJ, Coffey CS, Conwit R, et al. Phase 2 Trial of Ibudilast in Progressive Multiple Sclerosis. *New England Journal of Medicine* 2018;**379**(9):846-55.

For peer review only - http://bmjopen.bmj.com/site/about/guidelines.xhtml

21. Chan AW, Tetzlaff JM, Altman DG, et al. SPIRIT 2013 statement: defining standard protocol items for clinical trials. *Ann Intern Med* 2013;**158**(3):200-7.
22. Parker RA, Weir CJ. Non-adjustment for multiple testing in multi-arm trials of distinct treatments: Rationale and justification. *Clin Trials* 2020;**17**(5):562-66.
23. Choodari-Oskooei B, Bratton DJ, Gannon MR, et al. Adding new experimental arms to randomised clinical trials: Impact on error rates. *Clin Trials* 2020;**17**(3):273-84.
24. Odutayo A, Gryaznov D, Copsey B, et al. Design, analysis and reporting of multi-arm trials and strategies to address multiple testing. *Int J Epidemiol* 2020;**49**(3):968-78.
25. Wong C, Gregory JM, Liao J, et al. A Systematic Approach to Identify Neuroprotective Interventions for Motor Neuron Disease. *medRxiv* 2022:2022.04.13.22273823.
26. Wang R, Zhang D. Memantine prolongs survival in an amyotrophic lateral sclerosis mouse model. *European Journal of Neuroscience* 2005;**22**(9):2376-80.
27. de Carvalho M, Pinto S, Costa J, et al. A randomized, placebo-controlled trial of memantine for functional disability in amyotrophic lateral sclerosis. *Amyotrophic lateral sclerosis : official publication of the World Federation of Neurology Research Group on Motor Neuron Diseases* 2010;**11**(5):456-60.
28. Chan K, R T, G B, et al. C82 a randomized, double-blind, dose-ranging study of memantine in patients with amyotrophic lateral sclerosis. 22nd International Symposium on ALS/MND. *Amyotrophic Lateral Sclerosis* 2011;**12**(supp1):48.
29. Levine TD, Bowser R, Hank N, et al. A pilot trial of memantine and riluzole in ALS: correlation to CSF biomarkers. *Amyotroph Lateral Scler* 2010;**11**(6):514-9.
30. Halliday M, Radford H, Zents KAM, et al. Repurposed drugs targeting eIF2α-P-mediated translational repression prevent neurodegeneration in mice. *Brain* 2017;**140**(6):1768-83.
31. Kim HJ, Raphael AR, LaDow ES, et al. Therapeutic modulation of eIF2alpha phosphorylation rescues TDP-43 toxicity in amyotrophic lateral sclerosis disease models. *Nat Genet* 2014;**46**(2):152-60.
32. Lauterbach EC. Neuroprotective effects of psychotropic drugs in Huntington's disease. *Int J Mol Sci* 2013;**14**(11):22558-603.
33. Mehta AR, Gregory JM, Dando O, et al. Mitochondrial bioenergetic deficits in C9orf72 amyotrophic lateral sclerosis motor neurons cause dysfunctional axonal homeostasis. *Acta Neuropathol* 2021;**141**(2):257-79.
34. Lebert F, Stekke W, Hasenbroekx C, et al. Frontotemporal dementia: a randomised, controlled trial with trazodone. *Dement Geriatr Cogn Disord* 2004;**17**(4):355-9.
35. Cedarbaum JM, Stambler N, Malta E, et al. The ALSFRS-R: a revised ALS functional rating scale that incorporates assessments of respiratory function. BDNF ALS Study Group (Phase III). *Journal of the neurological sciences* 1999;**169**(1-2):13-21.
36. Kaufmann P, Levy G, Montes J, et al. Excellent inter-rater, intra-rater, and telephone-administered reliability of the ALSFRS-R in a multicenter clinical trial. *Amyotrophic lateral sclerosis : official publication of the World Federation of Neurology Research Group on Motor Neuron Diseases* 2007;**8**(1):42-6.
37. Atassi N, Berry J, Shui A, et al. The PRO-ACT database: design, initial analyses, and predictive features. *Neurology* 2014;**83**(19):1719-25.
38. PRO-ACT Open Access ALS Clinical Trial database Secondary PRO-ACT Open Access ALS Clinical Trial database http://www.alsdatabase.org/.
39. Clinical Trials Facilitation and Coordination Group. Recommendation Paper on the Initiation and Conduct of Complex Clinical Trials. .

For peer review only

FIGURES

Figure legends

Figure 1 Trial overview of MND-SMART

Figure 2 MND-SMART participant timeline. Boxes with red outline indicate study visits which can be conducted by telephone or secure videoconferencing. ALSFRS-R, Amyotrophic Lateral Sclerosis Functional Rating Scale Revised; CARE-MND, Clinical Audit Research Evaluation Motor Neuron Disease; ECAS, Edinburgh Cognitive and Behavioural ALS Screen; ECG, electrocardiogram; FVC, Forced Vital Capacity; HADS, Hospital Anxiety and Depression Scale.

For peer review only

For peer review only - http://bmjopen.bmj.com/site/about/guidelines.xhtml

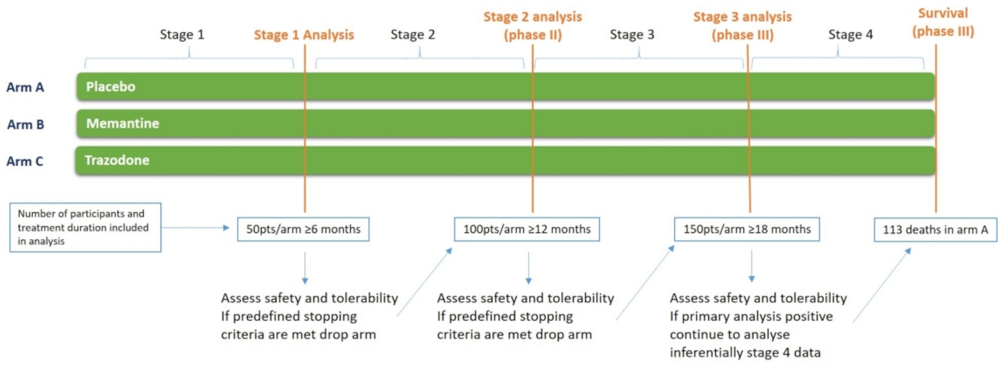

Trial overview of MND-SMART

326x121mm (300 x 300 DPI)

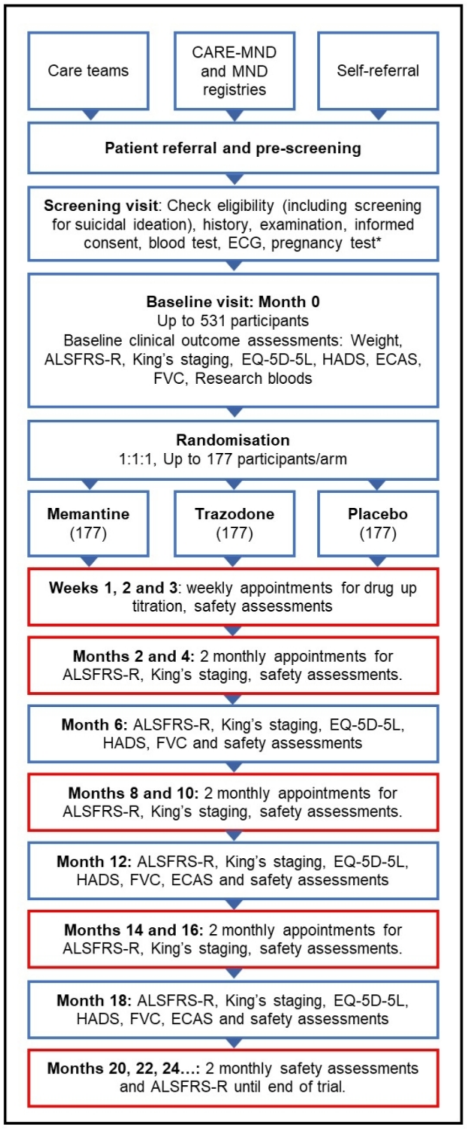

MND-SMART participant timeline. Boxes with red outline indicate study visits which can be conducted by telephone or secure videoconferencing. ALSFRS-R, Amyotrophic Lateral Sclerosis Functional Rating Scale Revised; CARE-MND, Clinical Audit Research Evaluation Motor Neuron Disease; ECAS, Edinburgh Cognitive and Behavioural ALS Screen; ECG, electrocardiogram; FVC, Forced Vital Capacity; HADS, Hospital Anxiety and Depression Scale.

70x150mm (300 x 300 DPI)

STANDARD PROTOCOL ITEMS: RECOMMENDATIONS FOR INTERVENTIONAL TRIALS

SPIRIT 2013 Checklist: Recommended items to address in a clinical trial protocol and related documents*

Section/item	ItemNo	Description
**Administrative information**		
Title	1	Descriptive title identifying the study design, population, interventions, and, if applicable, trial acronym<br><br>**Page 1**
Trial registration	2a	Trial identifier and registry name. If not yet registered, name of intended registry<br>**Page 2, 7**
	2b	All items from the World Health Organization Trial Registration Data Set<br>https://www.clinicaltrialsregister.eu/ctr-search/trial/2019-000099-41/GB<br>https://www.clinicaltrials.gov/ct2/show/NCT04302870
Protocol version	3	Date and version identifier<br>**Page 14; 24 and 25; Table 5**
Funding	4	Sources and types of financial, material, and other support<br>**Page 16**
Roles and responsibilities	5a	Names, affiliations, and roles of protocol contributors<br>**Page 1, 16**
	5b	Name and contact information for the trial sponsor<br>**Page 15**<br>https://www.clinicaltrialsregister.eu/ctr-search/trial/2019-000099-41/GB
	5c	Role of study sponsor and funders, if any, in study design; collection, management, analysis, and interpretation of data; writing of the report; and the decision to submit the report for publication, including whether they will have ultimate authority over any of these activities<br>**Page 16**

For peer review only

	5d	Composition, roles, and responsibilities of the coordinating centre, steering committee, endpoint adjudication committee, data management team, and other individuals or groups overseeing the trial, if applicable (see Item 21a for data monitoring committee)
**Introduction**		**Page 15**
Background and rationale	6a	Description of research question and justification for undertaking the trial, including summary of relevant studies (published and unpublished) examining benefits and harms for each intervention **Page 5, 6, 9**
	6b	Explanation for choice of comparators **Page 5, 6, 9**
Objectives	7	Specific objectives or hypotheses **Page 6, 7**
Trial design	8	Description of trial design including type of trial (eg, parallel group, crossover, factorial, single group), allocation ratio, and framework (eg, superiority, equivalence, noninferiority, exploratory) **Page 7, 8**
**Methods: Participants, interventions, and outcomes**		
Study setting	9	Description of study settings (eg, community clinic, academic hospital) and list of countries where data will be collected. Reference to where list of study sites can be obtained **Page 8**
Eligibility criteria	10	Inclusion and exclusion criteria for participants. If applicable, eligibility criteria for study centres and individuals who will perform the interventions (eg, surgeons, psychotherapists) **Page 8; Table 2**
Interventions	11a	Interventions for each group with sufficient detail to allow replication, including how and when they will be administered **Page 9; Table 3**
	11b	Criteria for discontinuing or modifying allocated interventions for a given trial participant (eg, drug dose change in response to harms, participant request, or improving/worsening disease) **Page 9**
	11c	Strategies to improve adherence to intervention protocols, and any procedures for monitoring adherence (eg, drug tablet return, laboratory tests) **Page 9**

For peer review only

	11d	Relevant concomitant care and interventions that are permitted or prohibited during the trial **Page 10**
Outcomes	12	Primary, secondary, and other outcomes, including the specific measurement variable (eg, systolic blood pressure), analysis metric (eg, change from baseline, final value, time to event), method of aggregation (eg, median, proportion), and time point for each outcome. Explanation of the clinical relevance of chosen efficacy and harm outcomes is strongly recommended **Page 10, 11, 12, 13**
Participant timeline	13	Time schedule of enrolment, interventions (including any run-ins and washouts), assessments, and visits for participants. A schematic diagram is highly recommended (see Figure) **Page 10; Table 4; Figure 2**
Sample size	14	Estimated number of participants needed to achieve study objectives and how it was determined, including clinical and statistical assumptions supporting any sample size calculations **Page 10, 11**
Recruitment	15	Strategies for achieving adequate participant enrolment to reach target sample size **Page 8**

Methods: Assignment of interventions (for controlled trials)

Allocation:

Sequence generation	16a	Method of generating the allocation sequence (eg, computer-generated random numbers), and list of any factors for stratification. To reduce predictability of a random sequence, details of any planned restriction (eg, blocking) should be provided in a separate document that is unavailable to those who enrol participants or assign interventions **Page 11**
Allocation concealment mechanism	16b	Mechanism of implementing the allocation sequence (eg, central telephone; sequentially numbered, opaque, sealed envelopes), describing any steps to conceal the sequence until interventions are assigned **Page 11**
Implementation	16c	Who will generate the allocation sequence, who will enrol participants, and who will assign participants to interventions **Page 11**

Blinding (masking)	17a	Who will be blinded after assignment to interventions (eg, trial participants, care providers, outcome assessors, data analysts), and how<br>**Page 11**
	17b	If blinded, circumstances under which unblinding is permissible, and procedure for revealing a participant's allocated intervention during the trial<br>**Page 11**
**Methods: Data collection, management, and analysis**		
Data collection methods	18a	Plans for assessment and collection of outcome, baseline, and other trial data, including any related processes to promote data quality (eg, duplicate measurements, training of assessors) and a description of study instruments (eg, questionnaires, laboratory tests) along with their reliability and validity, if known. Reference to where data collection forms can be found, if not in the protocol<br>**Page 10, 12**
	18b	Plans to promote participant retention and complete follow-up, including list of any outcome data to be collected for participants who discontinue or deviate from intervention protocols<br>**Page 7, 13, 14**
Data management	19	Plans for data entry, coding, security, and storage, including any related processes to promote data quality (eg, double data entry; range checks for data values). Reference to where details of data management procedures can be found, if not in the protocol<br>**Page 12**
Statistical methods	20a	Statistical methods for analysing primary and secondary outcomes. Reference to where other details of the statistical analysis plan can be found, if not in the protocol<br>**Page 11**
	20b	Methods for any additional analyses (eg, subgroup and adjusted analyses)<br>**Page 12, 13**
	20c	Definition of analysis population relating to protocol non-adherence (eg, as randomised analysis), and any statistical methods to handle missing data (eg, multiple imputation)<br>**Page 12, 13**
**Methods: Monitoring**		

Data monitoring	21a	Composition of data monitoring committee (DMC); summary of its role and reporting structure; statement of whether it is independent from the sponsor and competing interests; and reference to where further details about its charter can be found, if not in the protocol. Alternatively, an explanation of why a DMC is not needed **Page 13, 15**
	21b	Description of any interim analyses and stopping guidelines, including who will have access to these interim results and make the final decision to terminate the trial **Page 7, 13, 14**
Harms	22	Plans for collecting, assessing, reporting, and managing solicited and spontaneously reported adverse events and other unintended effects of trial interventions or trial conduct **Page 13**
Auditing	23	Frequency and procedures for auditing trial conduct, if any, and whether the process will be independent from investigators and the sponsor **Page 14**
**Ethics and dissemination**		
Research ethics approval	24	Plans for seeking research ethics committee/institutional review board (REC/IRB) approval **Page 14**
Protocol amendments	25	Plans for communicating important protocol modifications (eg, changes to eligibility criteria, outcomes, analyses) to relevant parties (eg, investigators, REC/IRBs, trial participants, trial registries, journals, regulators) **Page 14; Table 5**
Consent or assent	26a	Who will obtain informed consent or assent from potential trial participants or authorised surrogates, and how (see Item 32) **Page 8**
	26b	Additional consent provisions for collection and use of participant data and biological specimens in ancillary studies, if applicable
Confidentiality	27	How personal information about potential and enrolled participants will be collected, shared, and maintained in order to protect confidentiality before, during, and after the trial **Page 14, 15**
Declaration of interests	28	Financial and other competing interests for principal investigators for the overall trial and each study site **Page 16**

For peer review only

Access to data	29	Statement of who will have access to the final trial dataset, and disclosure of contractual agreements that limit such access for investigators<br>**Page 14**
Ancillary and post-trial care	30	Provisions, if any, for ancillary and post-trial care, and for compensation to those who suffer harm from trial participation
Dissemination policy	31a	Plans for investigators and sponsor to communicate trial results to participants, healthcare professionals, the public, and other relevant groups (eg, via publication, reporting in results databases, or other data sharing arrangements), including any publication restrictions<br>**Page 15**
	31b	Authorship eligibility guidelines and any intended use of professional writers
	31c	Plans, if any, for granting public access to the full protocol, participant-level dataset, and statistical code<br>**NA**
**Appendices**		
Informed consent materials	32	Model consent form and other related documentation given to participants and authorised surrogates<br>**Supplementary material**
Biological specimens	33	Plans for collection, laboratory evaluation, and storage of biological specimens for genetic or molecular analysis in the current trial and for future use in ancillary studies, if applicable

*It is strongly recommended that this checklist be read in conjunction with the SPIRIT 2013 Explanation & Elaboration for important clarification on the items. Amendments to the protocol should be tracked and dated. The SPIRIT checklist is copyrighted by the SPIRIT Group under the Creative Commons "Attribution-NonCommercial-NoDerivs 3.0 Unported" license.

For peer review only

BMJ Open

Motor Neuron Disease Systematic Multi-Arm Adaptive Randomised Trial (MND-SMART): A multi-arm, multi-stage, adaptive, platform, phase III randomised, double-blind, placebo-controlled trial of repurposed drugs in motor neuron disease.

Journal:	*BMJ Open*
Manuscript ID	bmjopen-2022-064173.R1
Article Type:	Protocol
Date Submitted by the Author:	11-May-2022
Complete List of Authors:	Wong, Charis; The University of Edinburgh Centre for Clinical Brain Sciences, Anne Rowling Regenerative Neurology Clinic; The University of Edinburgh Centre for Clinical Brain Sciences, Euan MacDonald Centre for Motor Neuron Disease Research<br>Dakin, Rachel; The University of Edinburgh Centre for Clinical Brain Sciences; The University of Edinburgh Centre for Clinical Brain Sciences, Anne Rowling Regenerative Neurology Clinic<br>Williamson, Jill; The University of Edinburgh Centre for Clinical Brain Sciences, Anne Rowling Regenerative Neurology Clinic; The University of Edinburgh Centre for Clinical Brain Sciences, Euan MacDonald Centre for Motor Neuron Disease Research<br>Newton, Judith; The University of Edinburgh Centre for Clinical Brain Sciences, Anne Rowling Regenerative Neurology Clinic; The University of Edinburgh Centre for Clinical Brain Sciences, Euan MacDonald Centre for Motor Neuron Disease Research<br>Steven, Michelle; The University of Edinburgh Usher Institute of Population Health Sciences and Informatics, Edinburgh Clinical Trials Unit<br>Colville, Shuna; The University of Edinburgh Centre for Clinical Brain Sciences, Anne Rowling Regenerative Neurology Clinic; The University of Edinburgh Centre for Clinical Brain Sciences, Euan MacDonald Centre for Motor Neuron Disease Research<br>Chataway, Jeremy; University College London Institute of Neurology Queen Square Multiple Sclerosis Centre, Neuroinflammation; National Institute for Health Research, University College London Hospitals<br>Swingler, Robert; The University of Edinburgh Centre for Clinical Brain Sciences, Euan MacDonald Centre for Motor Neuron Disease Research; London North West University Healthcare NHS Trust, Northwick Park Hospital<br>Parker, Richard; The University of Edinburgh Usher Institute of Population Health Sciences and Informatics, Edinburgh Clinical Trials Unit<br>Weir, Christopher; The University of Edinburgh Usher Institute of Population Health Sciences and Informatics, Edinburgh Clinical Trials Unit<br>Stallard, Nigel; University of Warwick, Statistics and Epidemiology, Division of Health Sciences, Warwick Medical School<br>Parmar, Mahesh; Medical Research Council Clinical Trials Unit at University College London, Institute of Clinical Trials and Methodology<br>Macleod, Malcolm; The University of Edinburgh Centre for Clinical Brain

	Sciences<br>Pal, Suvankar; The University of Edinburgh Centre for Clinical Brain Sciences, Anne Rowling Regenerative Neurology Clinic; The University of Edinburgh Centre for Clinical Brain Sciences, Euan MacDonald Centre for Motor Neuron Disease Research<br>Chandran, Siddharthan ; The University of Edinburgh Centre for Clinical Brain Sciences, Euan MacDonald Centre for Motor Neuron Disease Research; UK Dementia Research Institute, University of Edinburgh
**Primary Subject Heading**:	Neurology
Secondary Subject Heading:	Pharmacology and therapeutics
Keywords:	Motor neurone disease < NEUROLOGY, Clinical trials < THERAPEUTICS, Neurology < INTERNAL MEDICINE, STATISTICS & RESEARCH METHODS

SCHOLARONE™ Manuscripts

TITLE

Motor Neuron Disease Systematic Multi-Arm Adaptive Randomised Trial (MND-SMART): A multi-arm, multi-stage, adaptive, platform, phase III randomised, double-blind, placebo-controlled trial of repurposed drugs in motor neuron disease.

AUTHORS

Charis Wong[1,2,3]*, Rachel S Dakin[1,2,3]*, Jill Williamson[1,2,3], Judith Newton[1,2,3], Michelle Steven[4], Shuna Colville[1,2,3], Jeremy Chataway[5,6,7], Robert J Swingler[2,8], Richard A Parker[4], Christopher J Weir[4], Nigel Stallard[9], Mahesh KB Parmar[7], Malcolm R Macleod[1], Suvankar Pal[ψ1,2,3], Siddharthan Chandran[ψ1,2,3,10].

**Joint first authors*

ψCo-Corresponding authors:

Siddharthan Chandran PhD

Address: Centre for Clinical Brain Sciences, University of Edinburgh, Edinburgh, EH16 4SB, UK.

Email: Siddharthan.chandran@ed.ac.uk

Suvankar Pal

Address: Centre for Clinical Brain Sciences, University of Edinburgh, Edinburgh, EH16 4SB, UK.

Email: suvankar.pal@ed.ac.uk

Affiliations:

1. Centre of Clinical Brain Sciences, University of Edinburgh, Edinburgh, EH16 4SB, UK.
2. Euan Macdonald Centre for Motor Neuron Disease Research, University of Edinburgh, Edinburgh, EH16 4SB, UK.
3. Anne Rowling Regenerative Neurology Clinic, University of Edinburgh, Edinburgh, EH16 4SB, UK.
4. Edinburgh Clinical Trials Unit, Usher Institute of Population Health Sciences and Informatics, University of Edinburgh, Edinburgh, UK.
5. Queen Square Multiple Sclerosis Centre, Department of Neuroinflammation, UCL Queen Square Institute of Neurology, Faculty of Brain Sciences, University College London, London, United Kingdom WC1B 5EH
6. National Institute for Health Research, University College London Hospitals, Biomedical Research Centre, London, United Kingdom W1T 7DN
7. Medical Research Council Clinical Trials Unit at UCL, Institute of Clinical Trials and Methodology, University College London, London, United Kingdom WC1V 6LJ
8. London North West University Healthcare NHS Trust, Northwick Park Hospital, A404 Watford Rd, Harrow HA1 3UJ
9. Statistics and Epidemiology, Division of Health Sciences, Warwick Medical School, University of Warwick, Coventry, UK.
10. UK Dementia Research Institute Edinburgh, University of Edinburgh, Edinburgh, EH16 4SB, UK.

Word count: 3964 words

For peer review only

ORCID iD

Charis Wong 0000-0002-8488-037X

Rachel Dakin 0000-0002-3697-851

Jill Williamson 0000-0002-2263-5950

Jeremy Chataway 0000-0001-7286-6901

Richard Parker 0000-0002-2658-5022

Nigel Stallard 0000-0001-7781-1512

Mahesh Parmar 0000-0003-0166-1700

Malcolm R. Macleod 0000-0001-9187-9839

Suvankar Pal 0000-0003-4276-639X

Siddharthan Chandran 0000-0001-6827-1593

For peer review only

ABSTRACT

Introduction:

Motor neuron disease (MND) is a rapidly fatal neurodegenerative disease. Despite decades of research and clinical trials there remains no cure and only one globally approved drug, riluzole, which prolongs survival by 2-3 months. Recent improved mechanistic understanding of MND heralds a new translational era with many potential targets being identified that are ripe for clinical trials. Motor Neuron Disease Systematic Multi-Arm Adaptive Randomised Trial (MND-SMART) aims to evaluate the efficacy of drugs efficiently and definitively in a multi-arm, multi-stage, adaptive trial. The first two drugs selected for evaluation in MND-SMART are trazodone and memantine.

Methods and analysis:

Initially, up to 531 participants (177/arm) will be randomised 1:1:1 to oral liquid trazodone, memantine and placebo. The co-primary outcome measures are the Amyotrophic Lateral Sclerosis Functional Rating Scale Revised (ALSFRS-R) and survival. Comparisons will be conducted in four stages. The decision to continue randomising to arms after each stage will be made by the Trial Steering Committee who receive recommendations from the Independent Data Monitoring Committee. The primary analysis of ALSFRS-R will be conducted when 150 participants/arm, excluding long survivors, have completed 18 months of treatment; if positive the survival effect will be inferentially analysed when 113 deaths have been observed in the placebo group. The trial design ensures that other promising drugs can be added for evaluation in planned trial adaptations. Using this novel trial design reduces time, cost, and number of participants required to definitively (phase III) evaluate drugs and reduces exposure of participants to potentially ineffective treatments.

Ethics and dissemination

MND-SMART was approved by the West of Scotland Research Ethics Committee on 2 October 2019. (REC reference: 19/WS/0123) Results of the study will be submitted for publication in a peer-reviewed journal and a summary provided to participants.

Trial registration numbers

EudraCT Number: 2019-000099-41. ClinicalTrials.gov Identifier: NCT04302870.

Keywords: motor neuron disease, amyotrophic lateral sclerosis, randomised controlled trial, adaptive trials, multi-arm multi-stage trials.

For peer review only

STRENGTHS AND LIMITATIONS OF THIS STUDY

- The Motor Neuron Disease – Systematic Multi-Arm Adaptive Randomised Trial (MND-SMART) is the first multi-arm multi-stage (MAMS) phase III trial in motor neuron disease to launch in Europe.
- This novel trial design aims to achieve efficiencies in time, cost, and requirements for sample size through planned trial adaptations: ineffective arms can be dropped at interim analysis whilst new arms can be added.
- Broad inclusion criteria allow wider participation and ensures generalisability of results in a heterogenous condition.
- Drugs are selected using a combined approach of a data-driven framework incorporating published literature and expert opinion.
- The complexities of the conduct of this MAMS trial necessitate particular operational considerations.

For peer review only

INTRODUCTION

Motor neuron disease (MND) is an incurable, rapidly progressive and invariably fatal disease characterised by selective motor neuron loss. Despite 125 phase II to III drug trials between 2008 and 2019, riluzole, licensed in the UK by the National Institute for Health and Care Excellence (NICE) in 2001, remains the only disease-modifying drug with marketing authorisation in the UK and Europe, with a median survival benefit of 2-3 months.[1 ,2] Across the UK historical MND trial participation is less than 5%.[2] Recently, edaravone and masitinib have emerged as promising candidates following positive trials in enriched trial populations.[3 ,4] Edaravone was licensed in Japan and USA in 2015 and 2017 respectively. However, evidence of generalisable survival benefit for these drugs remain limited; in a long-term prospective cohort study, edaravone did not improve survival or slow functional decline.[5]

There are many challenges facing clinical trials for MND ranging from comparatively blunt outcome measures to disease factors including rapid progression and heterogeneity. Benchmark outcomes for MND trials are largely ordinal clinical rating scales and survival.[2 ,6] To detect a significant treatment effect using these measures, large sample sizes are often needed to achieve adequate study power, especially when using traditional fixed two-arm designs. To account for heterogeneity, many previous trials used strict inclusion criteria.[2 ,7] However, a mean exclusion rate of 60% in 38 previous trials did not reduce heterogeneity in survival or functional decline.[7] Strict inclusion criteria, diagnostic delay and the rapidly progressive and fatal nature of MND often results in a short time window for people with MND (pwMND) to participate in trials. Furthermore, trial retention is often poor – in a recent review, 40% of trials reported attrition rates ≥20%.[2] Adherence with trial protocol often becomes challenging with increasing disability. Movement, speech, swallowing and respiration are commonly affected, the latter causing some pwMND to be dependent on enteral feeding and ventilatory support. Cognitive and behavioural changes also occur in approximately 35% of pwMND with up to 15% having frontotemporal dementia (FTD).[8 ,9] Against this background, there have been major advances in our understanding of the pathobiology of MND as well as an increasing number of candidate medicines being identified supporting innovation in trial design for MND.

Multi-arm multi-stage (MAMS) and other adaptive platform trials have been used successfully in other fields, especially cancer medicine and infectious diseases, and are particularly effective where there are several promising medicines without clear *a priori* evidence to favour one over the other.[2 ,10 ,11] A multi-arm trial allows simultaneous evaluation of multiple treatments against a single control group. An adaptive trial design allows additional arms to be added in what is in effect a "continuous" trial platform. These features can provide major efficiency gains in time, cost and sample size requirements compared to running multiple (and almost certainly consecutive)

For peer review only

conventional two-arm studies, each with their own control arm. Beside MND-SMART trial, there are two other declared adaptive platform trials in MND: USA-based HEALEY amyotrophic lateral sclerosis Platform Trial[12] (ClinicalTrials.gov registration number NCT04297683) and European-based Treatment and Research Initiative to Cure amyotrophic lateral sclerosis 'MAGNET' platform trial.[13]

Drug repurposing has proven successful in many areas, including multiple sclerosis.[14] The lower development cost and shorter development timelines with availability of long-term safety data makes this a particularly attractive option for devastating conditions like MND where trial attrition rates are historically high.[2] Rapid advances in genomics, molecular and human stem cell technology has led to better understanding of the underlying pathobiology of MND and discovery of novel drug targets. Computational advances have made living systematic reviews of the entire published literature of MND and other neurodegenerative diseases which may share similar pathways feasible, thus providing a robust, comprehensive and continually updated evidence-base to identify, evaluate and prioritise candidate drugs for trials.[15-17] These principles were used to inform drug selection for the Multiple Sclerosis-Secondary Progressive Multi-Arm Randomisation Trial (MS-SMART), a multi-arm trial of repurposed drugs in progressive multiple sclerosis.[18 ,19] The initial candidate drugs for MS-SMART based on this approach were ibudilast, amiloride and riluzole, but due to drug supply issue, ibudilast was switched to fluoxetine. Although the drugs tested in MS-SMART did not show clear evidence of activity, ibudilast slowed progression of brain atrophy in a separate phase II trial.[20]

Against this background, Motor Neuron Disease – Systematic Multi-Arm Randomised Trial (MND-SMART) is a phase III randomised MAMS trial of repurposed drugs in MND. The first two candidate drugs to be tested are trazodone and memantine.

METHODS AND ANALYSIS

Trial objectives

The primary objective of MND-SMART is to assess the clinical effects of putative neuroprotective drugs compared to placebo as measured by rate of change in score of the Amyotrophic Lateral Sclerosis Functional Rating Scale Revised (ALSFRS-R), and conditional on identifying benefit on this, to assess the effect of these drugs on survival. Secondary objectives are to assess the safety profile of candidate drugs, and their effects on time to King's stage 4a (nutritional failure), time to King's stage 4b (respiratory failure), cognitive function and behaviour as assessed using the Edinburgh Cognitive and Behavioural ALS Screen (ECAS), respiratory function measured by forced

For peer review only

vital capacity (FVC), anxiety and depression measured by the Hospital Anxiety and Depression Scale (HADS), and quality of life evaluation using EQ-5D-5L.

Overview of design

MND-SMART is a phase III double-blind, randomised, placebo-controlled MAMS platform trial. MND-SMART will initially assess the efficacy of memantine (Arm A) and trazodone (Arm B) against a single contemporaneous placebo control arm (Arm C) with investigational medicinal products (IMPs) delivered as liquids. Participants are randomised 1:1:1 to Arms A, B and C with up to 531 pwMND (177/arm) randomised to complete the analysis of these arms (Figure 1). Allowing for early withdrawal and long survivors, this will give 150 participants/group in the primary analysis of ALSFRS-R. Patients will have five appointments in the first four to eight weeks followed by assessments every two months, most completed remotely, and two appointments for treatment cessation when this is required (Figure 2).

The ALSFRS-R comparisons will be conducted in three stages (Figure 1) with the opportunity to stop randomisation to arms that do not meet the predefined continuing criteria (Table 1) at the end of stages 1 of 2. Stage 1 will be completed when 50 participants/arm (excluding long survivors defined as >8 years since diagnosis at baseline) have completed 6 months of treatment and Stage 2 will be completed when 100 participants per arm (excluding long survivors) have completed 12 months of treatment. The primary analysis (stage 3) will be conducted based on rate of decline of ALSFRS-R when 150 participants/arm (excluding long survivors) complete 18 months of treatment. Participants will continue to take their allocated treatment until 113 deaths have been observed in the placebo group, or stage 3 analysis data is collected – whichever is later, with the primary analysis of survival being performed at this point.

For peer review only

Table 1 Timing and guidance for Stage 1 and Stage 2 analyses

**Stage**	**Number of pwALS/arm excluding long survivors**	**Criterion to continue to next stage**
Stage 1	50 with ≥6 months follow-up	95%CI of the rate of change in ALSFRS-R compared to placebo must include a relative improvement of 25% in the rate of decline
Stage 2	100 with ≥12 months follow-up	Improvement in rate of change in ALSFRS-R compared to placebo significant at pairwise one-sided 10% level

The study protocol follows the Standard Protocol Items: Recommendations for Interventional Trials guidelines[21] and was registered on the European Clinical Trials Registry (2019-000099-41) on 25th July 2019 and ClinicalTrials.gov (NCT04302870) on 10th March 2020.

Planned trial adaptations

In addition to stopping arms that do not meet continuing criteria at the end of stage 1 and 2, we intend to introduce new arms in the future by substantial amendment. Any new arm will be compared to contemporaneous controls with each comparison following the multi-stage process described above. As long as treatments are distinct, for example belonging to different classes of medicine, no multiplicity adjustments will be made in analysis of pairwise comparisons.[22-24]

Re-entry of participants into the trial

Participants will be able to enter the trial on more than once occasion, under certain conditions. To ensure a participant does not contribute data to more than one arm in the same treatment comparison, they will only be able to re-enter MND-SMART if they satisfy one of the following criteria: the arm they were randomised to has been dropped; they are in the control group and all experimental arms open at the time at which they were randomised have been dropped; or their data has been included in a final survival-based comparison. Any participant re-entering the trial will be screened for eligibility in the same way as a trial naïve individual. Previous trial entry will be noted to ensure their medical

For peer review only

and treatment history is linked to allow for long-term follow up of potential signals and understanding of investigational drugs exposed to.

Participants, interventions and outcomes

Trial setting

MND-SMART is recruiting across the UK with 17 recruiting centres (listed on mnd-smart.org) at 10th March 2022. Research staff will enter data on site via a secure electronic case report form (eCRF), with quality control and analysis conducted independently by Edinburgh Clinical Trials Unit (ECTU).

Eligibility criteria

Eligibility criteria for MND-SMART are shown in Table 2.

For peer review only

Table 2 MND-SMART eligibility criteria (protocol V 6.0). MND, Motor neuron disease; ALT, Alanine aminotransferase; ALP, alkaline phosphatase; GGT, gamma-glutamyltransferase; eGFR, estimated glomerular filtration rate; TSH, thyroid-stimulating hormone.

**Inclusion criteria**	
• Confirmed diagnosis of MND (including the following subtypes: Amyotrophic Lateral Sclerosis by El Escorial Criteria (possible, probable, and definite), Primary Lateral Sclerosis, and Progressive Muscular Atrophy).<br>• Over 18 years of age.<br>• Women of childbearing potential must have a negative pregnancy test within 7 days prior to, or at, the baseline visit.	• Women of childbearing potential and fertile men must be using an appropriate method of contraception to avoid any unlikely teratogenic effects of the selected drugs from time of consent, to 4 weeks after treatment inclusive.<br>• Willing and able to comply with the trial protocol and ability to understand and complete questionnaires.<br>• Written informed consent (this can be signed by a proxy in the case of limb dysfunction).
**Exclusion criteria**	
• Diagnosis of Frontotemporal Dementia (FTD-MND) or any other significant psychiatric disorder that prevents informed consent being given.<br>• Patients in the manic phase of bipolar disorder.<br>• Alcoholism (self-reported)<br>• Active suicide ideation assessed using the Columbia-Suicide Severity Rating Scale.<br>• On concurrent investigational medication (including biological therapy).<br>• Known hypersensitivity, including hereditary fructose intolerance, or adverse reaction to the active substances and their excipients or any past medical history contraindicating use of any of the investigational medicinal products (IMPs).<br>• Pregnancy or breast-feeding females.<br>• If ALT, ALP, bilirubin or GGT >3 times the upper limit of normal; creatinine clearance (creatinine clearance or eGFR) <30 ml/min; or serum free T4 >25pmol/l or TSH <0.2mU/l.	• If corrected QT interval on 12 lead ECG >450 ms.<br>• Diagnosis of ventricular arrhythmias, heart block or in the immediate recovery period after myocardial infarction (< 6 weeks).<br>• Already taking any of the IMPs in the protocol.<br>• Contraindications to any of the IMPs according to Summary of Product Characteristics (SPC).<br>• Taking a medication that interacts with the active substances and their excipients according to the SPCs, including but not limited to; Dextromethorphan, Amantadine; Ketamine, Monoamine-oxidase inhibitors ((MAOIs): Rasagiline, Selegiline, Safinamide, Tranylcypromine, Phenelzine, Isocarboxazid, Moclobemide).<br>• Patients who the PI considers will not be able to comply with the study protocol

For peer review only

Ascertainment and recruitment

Potential participants are identified through self-referral from potential participants who may be made aware of the trial by the media, literature or website (www.mnd-smart.org), by members of clinical and care teams, and using the existing National MND Care/Research platform (CARE-MND) in Scotland and other UK wide MND registries that contain contact details of people who have consented to be contacted directly about relevant research opportunities. Potential participants will be informed about the study and given a patient information sheet (PIS). They can be pre-screened by the individual's clinical team or, in Scotland, against trial criteria using information from CARE-MND.

The screening visit includes documentation of written informed consent. If a participant is physically unable to give written consent due to limb dysfunction, verbal consent can be given with a witness independent of the trial signing the consent form. A clinical trial physician will evaluate each potential participant against the trial eligibility criteria and recruit participants. Each centre is required to maintain an anonymised log of all ineligible patients and eligible patients who declined participation.

The first participant was randomised on 27th February 2020.

Identifying the research interventions for priority evaluation in MND-SMART

For peer review only

We conducted a two-stage systematic review of the clinical literature of MND and four other neurodegenerative diseases (Alzheimer's disease, Parkinson's disease, Huntington's disease, and Multiple Sclerosis) which may share similar pathways, and of the preclinical MND literature to inform identification and selection of investigational drugs for MND-SMART by an expert panel.[25]Through this structured process memantine and trazodone were selected for evaluation. Memantine is a non-competitive N-methyl-D-aspartate (NMDA) receptor antagonist used in the management of Alzheimer's disease. It was shown to significantly delay disease progression and improve survival in mouse models carrying a high copy number of $SOD1^{G93A}$.[26] Three early phase MND clinical trials using memantine showed some evidence of safety and potential signals for efficacy in ALSFRS-R and in cerebrospinal and neurophysiological biomarkers.[27-29] Trazodone is an atypical antidepressant drug of the serotonin antagonist and reuptake inhibitor class. In an unbiased drug screen, trazodone inhibited Protein Kinase RNA-like endoplasmic reticulum kinase (PERK), which is pivotal to stress granule formation, a common feature of neurodegenerative diseases.[30] Inhibition of PERK was found to be beneficial in an ALS fly model as well as in an *in vitro* neuronal assay of TDP-43 injury.[31] Modulation of ER-stress response by trazodone resulted in improvement in survival of prion disease and Frontotemporal dementia (FTD) animal models.[30] In Huntington's disease mouse models, trazodone improved behavioural outcomes and exerted mitochondrial protective effects,[32] a pathway of interest in MND.[33] In a phase II trial in 31 people with FTD, trazodone improved cognition as assessed by the neuropsychiatric inventory.[34]

Drug allocation, titration and monitoring

Participants are randomised to memantine hydrochloride 10 mg/5 ml oral solution, trazodone hydrochloride 100 mg/5 ml oral solution or placebo oral solution containing no active pharmaceutical ingredient via a web-based randomisation system to maintain allocation concealment. Drugs and placebo are manufactured by Huddersfield Pharmacy Specials as liquids with identical colour, flavour and consistency. Weekly, during the first 4 weeks of the study, a clinical trial physician assesses participants for adverse events (AEs) and tolerability. IMP dose is also titrated using the dosing schedule shown in Table 3. Participants are asked to record adherence, drug doses and any reasons for non-adherence.

For peer review only

Table 3 MND-SMART dose titration schedule

	Baseline (Week 0) 'quarter dose': 2.5 ml	Week 1 'half dose': 5 ml	Week 2 'three quarter dose': 7.5 ml	Week 3 'full dose': 10 ml
Memantine	5 mg	10 mg	15 mg	20 mg
Trazodone	50 mg	100 mg	150 mg	200 mg
Placebo	2.5 ml	5 ml	7.5 ml	10 ml

Participants may stop treatment early with appropriate down-titration due to unacceptable AEs, pregnancy, inter-current illness that prevents further treatment, and any changes in the participant's condition justifying discontinuation of treatment according to investigators' discretion, including as part of end-of-life care.

Concomitant care and interventions

All concomitant medications are permitted except dextromethorphan, amantadine, ketamine, rasagiline, selegiline, safinamide, tranylcypromine, phenelzine, isocarboxazid and moclobemide. Participants are allowed to start standard of care, including riluzole and interventions like gastrostomy and non-invasive ventilation (NIV), after randomisation.

Outcome measures

Primary outcome measures

ALSFRS-R and survival are co-primary outcomes in MND-SMART. ALSFRS-R assesses MND-related disability in gross and fine motor tasks, bulbar function and respiratory status.[35] This scale has been widely used and validated as an appropriate and accurate assessment of functional ability and disease progression. It is easy to administer and has been validated for administration in person or over the telephone or by videoconferencing with high inter-rater and intra-rater reliability.[36]

Secondary outcomes measures

Secondary outcomes for MND-SMART include time to King's stage 4a and time to King's stage 4b, cognitive function and behaviour as assessed using ECAS, FVC, HADS and EQ-5D-5L quality of life evaluation.

Exploratory outcome

For peer review only

An exploratory outcome investigating ALSFRS-R scores post 18 months participation is also included.

Safety and tolerability outcomes

All study visits will capture data on AEs and adherence.

Participant timeline

Participant timeline and visit schedule is shown in Figure 2 and Table 4. Most study visits can be conducted by telephone or secure videoconferencing.

Table 4 MND-SMART visit and assessment schedule. [A]These appointments can be completed using video conferencing or telephone call. [B] These appointments should be completed face to face wherever possible. * For women of childbearing potential. † If more than 7 days from previous test. ¥ modified King's staging will take place at remote appointments ✓ start IMP. ‡ this assessment includes an interview with a partner/relative/friend/carer if the participant has consented. If an arm is dropped the IMP dose should be halved at the next scheduled appointment and participants then complete the two treatment cessation appointments. Appointments from 15 onwards are the same to be repeated every 2 months

For peer review only

**Appointment**	**Time**	Informed consent	Inclusion/exclusion review	Eligibility confirmation	Demography	Disease history	Past medical history	Current medications	Safety bloods	ECG	Suicide ideation (CSSRS)	Pregnancy test	Randomisation	FVC	ALSFRS-R & King's staging¥	EQ-5D-5L	HADS	ECAS ‡	Sub-studies	Research bloods	Drug supply	Titration up	Compliance check	Adverse Events
**1 Screening**		x	x	x	x	x	x	x	x	x	x	x*							x					x
**2 Baseline**	**Time 0**		x	x				x				x†	x	x	x	x	x	x	x	x	x	✓		x
**3, 4, 5 [A]**	**Weeks 1, 2 and 3**							x														x	x	x
**6, 7, 9, 10, 12, 13 [A]**	**Month 2, 4, 8, 10, 14, 16**							x							x				x		x		x	x
**8 [B]**	**Month 6**							x						x	x	x	x		x	x	x		x	x
**11 [B]**	**Month 12**							x						x	x	x	x	x	x	x	x		x	x
**14 [B]**	**Month 18**							x						x	x	x	x	x	x	x	x		x	x
**15, 16, 17… [A]**	**Month 20, 22, 24…**							x							x						x		x	x

For peer review only

Number of Patients required

For sample size calculations, simulation studies were performed using data from Pooled Resource Open-Access ALS Clinical Trials (PRO-ACT) Open Access ALS Clinical Trial database which pooled data from several MND clinical trials.[37 ,38] The simulations included interim analyses at the end of stages 1 and 2. For arms continuing beyond the second stage, the primary analysis was simulated based on 18-month follow-up data for 150 participants/arm excluding long survivors. This primary analysis plan is described in the Statistical Methods section below, and it should be noted that the design is calculated controlling the pairwise error rate at 2.5% (one-sided). To allow for a drop-out rate of approximately 10%, and for the exclusion from the analysis of long survivors (estimated to make up 5% of the trial population), we will randomise 177 participants to each treatment arm (total 531).

The simulations demonstrated that for a true 25% reduction in the rate of decline of ALSFRS-R, the sample size is sufficient to give a probability of 86% of continuing beyond the stage 2 analysis into the final stage and a probability of continuing to the final stage and obtaining a significant result of 83%, equivalent to an independent phase III trial power of 90%.The probability of dropping an active arm at the first interim analysis is 23% if the true treatment effect is identical to the control group and 2% if there is a true 25% reduction in the average decrease in ALSFRS-R in that arm.

With regards to the survival co-primary outcome, follow-up will continue until 113 deaths have been observed in the placebo arm. With 113 deaths/arm, this test will have 90% power to detect a hazard ratio of 0.65. To maintain overall type I error control at 2.5% (one-sided), survival will be analysed inferentially for an IMP only if statistically significant benefit has been demonstrated on ALSFRS-R. In the situation that the ALSFRS-R outcome measure does not produce a statistically significant result overall survival data will be analysed to provide supporting information but will not provide pivotal evidence of efficacy.

Assignment of interventions

Treatment allocation and blinding

Randomisation is performed at baseline visit via a secure web-based randomisation system employing the following minimisation variables: riluzole use, use of NIV and/or gastrostomy, and long survival (>8 years from diagnosis). The allocation sequence incorporates a random element based on computer-generated pseudo-random numbers to maintain unpredictability. Participants, investigators, care providers and outcome assessors are blinded to treatment allocation. To maintain blinding, each bottle is identified by a unique bottle number. In situations where medical management necessitates

For peer review only

For peer review only - http://bmjopen.bmj.com/site/about/guidelines.xhtml

knowledge of the study medication, emergency unblinding can be done through the web-based randomisation system.

Data Collection, management and analysis

Data collection and management

Data is collected by trained site staff and entered into the eCRF via a web-based portal. Access to the eCRF is role-based and delegated site staff are assigned unique usernames. Activity on the eCRF is tracked and auditable. The trial database includes in-built measures to reduce data entry errors and ensure data quality. Regular central data checks are performed with queries and follow up undertaken on a site by site basis. The eCRF is hosted by the University of Edinburgh in their secure environment as detailed in the trial data management plan.

Statistical methods

A CONSORT flow diagram will be reported. Descriptive statistics will be used to summarise baseline characteristics for each treatment group. Participants will be included in the analysis if they were not long survivors at baseline and have at least one ALSFRS-R score recorded either at baseline or during follow-up.

A hierarchical normal linear model will be used to compare the rate of decrease in ALSFRS-R over the follow-up period in each of the two active treatment group arms with the placebo arm. The analysis will adjust for the effects of baseline minimisation variables. Long survivors at baseline are not included in the primary ALSFRS-R analysis and so adjustment for this minimisation variable is not required. Estimates and 95% confidence intervals will be obtained for the difference in ALSFRS-R decrease for each active treatment group minus placebo. At the first interim analysis, each experimental arm will continue to the second stage if the upper limit of its 95% confidence interval for the rate of change of ALSFRS-R compared to placebo lies below a 25% reduction in the rate of decline. For example, a 95% confidence interval for reduction in rate of decline of (-10%, 30%) would lead to continuation of the experimental arm, whereas the interval (-20%, 15%) would result in discontinuation.

At the second interim analysis, if a significant treatment benefit relative to the placebo is observed at the pairwise two-sided 20% significance level, then the treatment will proceed to the final (end of stage 3) analysis. At the final analysis, each pairwise comparison of trazodone and memantine with placebo, will be tested using a one-sided 2.5% significance. Most types of intercurrent events will be handled in the estimand using the treatment policy strategy, except for deaths which will be handled

For peer review only - http://bmjopen.bmj.com/site/about/guidelines.xhtml

using the hypothetical strategy. A detailed statistical analysis plan will contain full information on the estimands for ALSFRS-R and survival.

Analysis of the survival co-primary outcome at the end of stage 4 will be based on all participants, including long survivors at baseline. Kaplan Meier statistics will estimate the survival function and the median survival will be calculated for each group with 95% confidence intervals. Log-rank tests will be used to compare treatment and placebo survival curves and treatment effects will be estimated by a Cox proportional hazard model including covariates to allow for the effects of the baseline minimisation variables.

Pre-planned subgroup analyses of primary and secondary outcome measures will be performed for: participants with different survival at baseline (>8 years, < 8 years); participants with different subtypes of MND (amyotrophic lateral sclerosis, primary lateral sclerosis and progressive muscular atrophy); participants with C9orf72 expansions; participants with bulbar-onset and limb-onset forms of MND; participants age <40 or age >80; participants receiving support with NIV at randomisation and participants receiving feeding support with gastrostomy at randomisation..

Missing data handling methods will be described in the full statistical analysis plan.

Monitoring

Data monitoring

An Independent Data Monitoring Committee (IDMC) oversees the safety of participants in the trial and review interim analyses. The committee regularly monitors safety data and makes recommendations on whether the trial should continue as planned and on the dropping of treatment arms following formal interim analyses. The committee is composed of independent members, without competing interests, and meetings are conducted in accordance with the IDMC charter.

Adverse Events (AEs)

Detection, recording, and reporting adverse events

Participants are asked about the occurrence of AEs at every study appointment. AE data is also available from participant diaries and laboratory results. Each AE will be assessed at site for seriousness, causality and severity. A central unblinded team assesses the expectedness of all adverse reactions and serious adverse reactions. AEs due to pre-existing medical conditions are only reported if medically judged to be an unexpected worsening during the study. AEs related to MND of a participant should not be recorded for the trial if consistent with disease progression.

For peer review only - http://bmjopen.bmj.com/site/about/guidelines.xhtml

For peer review only

Withdrawal of study participants and early termination of trial

Participants are free to withdraw from the study at any point or a participant can be withdrawn by investigators. If withdrawal occurs, the primary reason will be documented in the participant's case record form. Discontinuing study drug does not constitute withdrawal – all attempts should be made to follow up the participant as per protocol and/or to re-commence treatment (if discontinuation was due to AEs which have subsequently resolved). Participants can choose to stop all appointments but remain in the trial for follow-up of survival from medical records only. Withdrawn subjects will not be replaced. The IDMC can request trial arm suspension or termination, for example due to unacceptable AEs.

Auditing

University of Edinburgh and NHS Lothian Academic and Clinical Central Office for Research and Development (ACCORD) is the trial sponsor. ACCORD personnel, or designees, will perform monitoring activities and study audits in accordance with the trial monitoring plan. Data is continually audited by the central team for return rate and completeness.

Patient and public involvement

Patient public involvement (PPI) has been crucial throughout the development of MND-SMART. Through support groups and a PPI advisory group, pwMND, their families and carers were consulted and provided suggestions to optimise trial recruitment and retention. These included limiting number of appointments or travel to appointments, use of remote appointments supported by telehealth, delivery of IMP to participants' homes where necessary, use of liquid IMP formulations to enable participants with swallowing difficulties including those using gastrostomy to participate and remain in trial, and using broad inclusion criteria where the main exclusions are limited to contraindications to study drugs and lack of capacity to consent.

ETHICS AND DISSEMINATION

Research ethics approval

MND-SMART was approved by the West of Scotland Research Ethics Committee on 2 October 2019. (REC reference: 19/WS/0123).

Protocol amendments

Protocol versions and substantial amendments to date are summarised in Table 5.

For peer review only

Table 5 Protocol versions and substantial amendments to date.* protocol v2.0 was given CTA

Protocol update	Protocol version	Protocol date	Reason for amendment
NA	V1	11th July 2019	N/A
Substantial amendment	V2*	20th August 2019	Addition of Clinical Trials Coordination and Facilitation Group (CTFG) definition of women of childbearing potential; and information on highly effective contraceptive methods. Additional pregnancy test added at the end of systemic exposure for women of childbearing potential
Substantial amendment – SA01	V3	24th October 2019	Clarification on trial design:<br>- Treatment and follow up duration will continue until 200 patients/group have 18 months follow up (whichever is later)<br><br>Guidance on prioritising primary endpoints, completing modified King's staging in remote appointments and timescale for completing carer part of the ECAS, within 2 weeks of participant part of assessment.<br><br>Addition that adverse events that are consistent with pre-existing medical conditions and/or the progression of MND do not need to be recorded in the CRF or reported (if serious) but should be noted in medical records.<br><br>Confirmation of the procedure for assigning expectedness to adverse reactions by the central expectedness team.
Substantial amendment – SA02	V3	24th October 2019	Addition of site
Substantial amendment – SA03	V3	24th October 2019	Addition of sites
Substantial amendment – SA04	V3	24th October 2019	Change of PI
Substantial amendment – SA05	V5	1st March 2021	Updates to exclusion criteria<br>Confirmation on pre-screening process and addition of remote consent using electronic software.<br>Update to allow use of clinical results for eligibility review and outcome assessments

For peer review only - http://bmjopen.bmj.com/site/about/guidelines.xhtml

			and addition of possibility for CI to unblind. Confirmation patients who withdraw can consent to follow-up of survival from medical records. Addition of ability to cross cover for sites and updates to requirements to courier drugs direct to participants including removing need to return IMP bottles to site for compliance. Addition of sending patient reported outcome questionnaires direct by email and clarification on assessments which can be performed remotely.
Substantial amendment – SA06	V5	1st March 2021	Addition of sites
Substantial amendment – SA07	V6	1st November 2021	Update to sample size based on move from family wise to pair wise Type I error control. Removal of SNIP (sniff nasal inspiratory pressure) as a secondary outcome measure. Confirmation removal of IMPs from trial does not constitute study end, new IMPs will be added. Addition of exploratory objective to investigate ALS_FRS-R post 18months participation. Clarifications on consent for patients with limb dysfunction, co-enrolment, IMP discontinuation and other points.

Confidentiality and access to data

All laboratory specimens, evaluation forms, reports, and other records are identified with as little personal information as possible to maintain participant confidentiality. Access to personal information is restricted to individuals from the research team treating the participants, representatives of the Sponsor(s) and regulatory authorities. Those analysing samples or data will not have access to personal information. The trial master file is held by the Anne Rowling Clinic and the database is held by ECTU for a minimum of 25 years from the defined end of study point.

Dissemination policy

To ensure data transparency, the results of any closed comparisons will be published in a peer reviewed journal as soon as it is possible when the integrity of the trial will not be affected.[39] Result of all analyses will be published. To maintain the scientific integrity of the study, data will not be released prior to the first publication of the results of the primary endpoint analysis, without the permission of the Trial Steering Committee (TSC). The TSC will agree a publication plan and must be consulted prior to release or publication of any study data. A summary of results will be provided to all participants via newsletters and the trial website.

For peer review only

ACKNOWLEDGMENTS

The authors would like to thank all the MND-SMART participants and collaborators involved in the trial. Particular thanks to the trial public patient involvement group who informed trial design and patient facing documents. The authors would like to thank the members of the Independent Data Monitoring Committee: Stephen Wroe (Chair), Camille Caroll, Sue Todd; and the members of the Trial Steering Committee: Helen Ford (Chair), Hadi Manji, James Carpenter, Beverley Gray, and former member Alan Gray. Additional thanks to Ronnie Harkess for designing the trial database and Catriona Keerie for statistical analysis. Particularly thanks also to all research nurses and practitioners involved in the trial.

MND-SMART is co-sponsored by University of Edinburgh and NHS Lothian Academic and Clinical Central Office for Research and Development (ACCORD).

The current MND-SMART sites and investigators are; Aberdeen Royal Cornhill Hospital, NHS Grampian, Dr Callum Duncan; Barts Health NHS Trust, Royal London Hospital, Dr Aleksandar Radunovic; Birmingham Queen Elizabeth Hospital, University Hospitals Birmingham NHS Foundation Trust, Dr Venkataramanan Srinivasan; Cambridge University Hospitals NHS Foundation Trust, Dr Rhys Roberts; Cardiff and Value University Health Board, University Hospital Wales, Dr Ken Dawson; Dundee Ninewells Hospital, NHS Tayside, Dr Ian Morrison, Edinburgh Anne Rowling Regenerative Neurology Clinic, NHS Lothian, Dr Suvankar Pal, Royal Devon and Exeter NHS Foundation Trust, Dr Timothy Harrower, Glasgow Queen Elizabeth University Hospital, NHS Greater Glasgow and Clyde, Dr George Gorrie, Inverness Raigmore Hospital, NHS Highland, Dr Javier Carod-Artal, Ipswich Hospital, East Suffolk and North Essex NHS Foundation Trust, Dr Clare Galton, Newcastle MND Care and Research Centre, Newcastle upon Tyne Hospitals NHS Foundation Trust, Dr Timothy Williams; Norfolk and Norwich University Hospitals NHS Foundation Trust, Dr Godwin Mamutse; Salford Royal NHS Foundation Trust, Dr Hisham Hamdalla, University Hospital Southampton NHS Foundation Trust, Dr Ashwin Pinto, St George's University Hospital NHS Foundation Trust, Dr Pablo Garcia Reitboeck; West Suffolk NHS Foundation Trust, Dr Francesca Crawley.

AUTHORS' CONTRIBUTIONS:

SCh is the chief investigator and provides clinical expertise and with SP and MKBP is responsible for study concept and design. CW, JN, JC, MM and RJS provide clinical and scientific expertise and helped with design of the study. MM and SCh conceptualised the drug selection strategy. RAP, CJW, NS and MKBP provided statistical expertise for trial design. RAP and CJW are the trial statisticians and provide expertise in day to day running of the trial. RSD, JW and SCo contributed to study design and trial management. MS is the data manager and provided expertise for trial design and day to day management. RSD is the trial manager and responsible for the running of the trial. CW and RSD contributed equally to this paper.

For peer review only - http://bmjopen.bmj.com/site/about/guidelines.xhtml

FUNDING STATEMENT

MND-SMART is funded by grants from MND Scotland, My Name'5 Doddie Foundation (DOD/14/15) and specific donations to the Euan MacDonald Centre. CJW, RAP and MS were supported in this work by NHS Lothian via Edinburgh Clinical Trials Unit. SCh receives funding from UK Research and Innovation (Medical Research Council) (DRI-CORE-2017-EDI). The study funders have no role in study design, data collection, data analysis, data interpretation, or writing of publications. For the purpose of open access, the author has applied a Creative Commons Attribution (CC BY) licence to any Author Accepted Manuscript version arising from this submission.

COMPETING INTERESTS STATEMENT

CJW is a member of the independent data monitoring committee for a trial of masitinib in ALS, for which his institution receives funding from AB Science.

In the last 3 years, JC has received support from the Efficacy and Evaluation (EME) Programme, a Medical Research Council (MRC) and National Institute for Health Research (NIHR) partnership and the Health Technology Assessment (HTA) Programme (NIHR), the UK MS Society, the US National MS Society and the Rosetrees Trust. He is supported in part by the NIHR University College London Hospitals (UCLH) Biomedical Research Centre, London, UK. He has been a local principal investigator for a trial in MS funded by the Canadian MS society. A local principal investigator for commercial trials funded by: Actelion, Novartis and Roche; and has taken part in advisory boards/consultancy for Azadyne, Janssen, Merck, NervGen, Novartis and Roche.

For peer review only

REFERENCES

1. Miller RG, Mitchell JD, Lyon M, et al. Riluzole for amyotrophic lateral sclerosis (ALS)/motor neuron disease (MND). *Cochrane Database Syst Rev* 2002(2):CD001447.
2. Wong C, Stavrou M, Elliott E, et al. Clinical trials in amyotrophic lateral sclerosis: a systematic review and perspective. *Brain Commun* 2021;**3**(4):fcab242.
3. Mora JS, Genge A, Chio A, et al. Masitinib as an add-on therapy to riluzole in patients with amyotrophic lateral sclerosis: a randomized clinical trial. *Amyotroph Lateral Scler Frontotemporal Degener* 2020;**21**(1-2):5-14.
4. Safety and efficacy of edaravone in well defined patients with amyotrophic lateral sclerosis: a randomised, double-blind, placebo-controlled trial. *Lancet Neurol* 2017;**16**(7):505-12.
5. Witzel S, Maier A, Steinbach R, et al. Safety and Effectiveness of Long-term Intravenous Administration of Edaravone for Treatment of Patients With Amyotrophic Lateral Sclerosis. *JAMA Neurol* 2022.
6. van den Berg LH, Sorenson E, Gronseth G, et al. Revised Airlie House consensus guidelines for design and implementation of ALS clinical trials. *Neurology* 2019;**92**(14):e1610-e23.
7. van Eijk RPA, Westeneng HJ, Nikolakopoulos S, et al. Refining eligibility criteria for amyotrophic lateral sclerosis clinical trials. *Neurology* 2019;**92**(5):e451-60.
8. Crockford C, Newton J, Lonergan K, et al. ALS-specific cognitive and behavior changes associated with advancing disease stage in ALS. *Neurology* 2018;**91**(15):e1370.
9. Phukan J, Elamin M, Bede P, et al. The syndrome of cognitive impairment in amyotrophic lateral sclerosis: a population-based study. *Journal of Neurology, Neurosurgery & Psychiatry* 2012;**83**(1):102.
10. Parmar MK, Sydes MR, Cafferty FH, et al. Testing many treatments within a single protocol over 10 years at MRC Clinical Trials Unit at UCL: Multi-arm, multi-stage platform, umbrella and basket protocols. *Clin Trials* 2017;**14**(5):451-61.
11. Mehta AR, Chataway J, Pal S, et al. Trials for neurodegenerative diseases: time to innovate. *Lancet Neurol* 2021;**20**(12):984.
12. Paganoni S, Berry JD, Quintana M, et al. Adaptive Platform Trials to Transform Amyotrophic Lateral Sclerosis Therapy Development. *Ann Neurol* 2022;**91**(2):165-75.
13. van Eijk RPA, Kliest T, McDermott CJ, et al. TRICALS: creating a highway toward a cure. *Amyotroph Lateral Scler Frontotemporal Degener* 2020;**21**(7-8):496-501.
14. Pushpakom S, Iorio F, Eyers PA, et al. Drug repurposing: progress, challenges and recommendations. *Nat Rev Drug Discov* 2019;**18**(1):41-58.
15. Bannach-Brown A, Przybyla P, Thomas J, et al. Machine learning algorithms for systematic review: reducing workload in a preclinical review of animal studies and reducing human screening error. *Systematic reviews* 2019;**8**(1):23.
16. Elliott JH, Synnot A, Turner T, et al. Living systematic review: 1. Introduction-the why, what, when, and how. *Journal of clinical epidemiology* 2017;**91**:23-30.
17. Wong C, Liao J, Gregory J, et al. Study protocol: ReLiSyR-MND (Repurposing Living Systematic Review – Motor Neuron Disease): A systematic approach to identify neuroprotective interventions for motor neuron disease Secondary Study protocol: ReLiSyR-MND (Repurposing Living Systematic Review – Motor Neuron Disease): A systematic approach to identify neuroprotective interventions for motor neuron disease 2021. https://osf.io/5jp43.
18. Connick P, De Angelis F, Parker RA, et al. Multiple Sclerosis-Secondary Progressive Multi-Arm Randomisation Trial (MS-SMART): a multiarm phase IIb randomised, double-blind, placebo-controlled clinical trial comparing the efficacy of three neuroprotective drugs in secondary progressive multiple sclerosis. *BMJ open* 2018;**8**(8):e021944.
19. Vesterinen HM, Connick P, Irvine CM, et al. Drug repurposing: a systematic approach to evaluate candidate oral neuroprotective interventions for secondary progressive multiple sclerosis. *PloS one* 2015;**10**(4):e0117705.
20. Fox RJ, Coffey CS, Conwit R, et al. Phase 2 Trial of Ibudilast in Progressive Multiple Sclerosis. *New England Journal of Medicine* 2018;**379**(9):846-55.

For peer review only - http://bmjopen.bmj.com/site/about/guidelines.xhtml

21. Chan AW, Tetzlaff JM, Altman DG, et al. SPIRIT 2013 statement: defining standard protocol items for clinical trials. *Ann Intern Med* 2013;**158**(3):200-7.
22. Parker RA, Weir CJ. Non-adjustment for multiple testing in multi-arm trials of distinct treatments: Rationale and justification. *Clin Trials* 2020;**17**(5):562-66.
23. Choodari-Oskooei B, Bratton DJ, Gannon MR, et al. Adding new experimental arms to randomised clinical trials: Impact on error rates. *Clin Trials* 2020;**17**(3):273-84.
24. Odutayo A, Gryaznov D, Copsey B, et al. Design, analysis and reporting of multi-arm trials and strategies to address multiple testing. *Int J Epidemiol* 2020;**49**(3):968-78.
25. Wong C, Gregory JM, Liao J, et al. A Systematic Approach to Identify Neuroprotective Interventions for Motor Neuron Disease. *medRxiv* 2022:2022.04.13.22273823.
26. Wang R, Zhang D. Memantine prolongs survival in an amyotrophic lateral sclerosis mouse model. *European Journal of Neuroscience* 2005;**22**(9):2376-80.
27. de Carvalho M, Pinto S, Costa J, et al. A randomized, placebo-controlled trial of memantine for functional disability in amyotrophic lateral sclerosis. *Amyotrophic lateral sclerosis : official publication of the World Federation of Neurology Research Group on Motor Neuron Diseases* 2010;**11**(5):456-60.
28. Chan K, R T, G B, et al. C82 a randomized, double-blind, dose-ranging study of memantine in patients with amyotrophic lateral sclerosis. 22nd International Symposium on ALS/MND. *Amyotrophic Lateral Sclerosis* 2011;**12**(supp1):48.
29. Levine TD, Bowser R, Hank N, et al. A pilot trial of memantine and riluzole in ALS: correlation to CSF biomarkers. *Amyotroph Lateral Scler* 2010;**11**(6):514-9.
30. Halliday M, Radford H, Zents KAM, et al. Repurposed drugs targeting eIF2α-P-mediated translational repression prevent neurodegeneration in mice. *Brain* 2017;**140**(6):1768-83.
31. Kim HJ, Raphael AR, LaDow ES, et al. Therapeutic modulation of eIF2alpha phosphorylation rescues TDP-43 toxicity in amyotrophic lateral sclerosis disease models. *Nat Genet* 2014;**46**(2):152-60.
32. Lauterbach EC. Neuroprotective effects of psychotropic drugs in Huntington's disease. *Int J Mol Sci* 2013;**14**(11):22558-603.
33. Mehta AR, Gregory JM, Dando O, et al. Mitochondrial bioenergetic deficits in C9orf72 amyotrophic lateral sclerosis motor neurons cause dysfunctional axonal homeostasis. *Acta Neuropathol* 2021;**141**(2):257-79.
34. Lebert F, Stekke W, Hasenbroekx C, et al. Frontotemporal dementia: a randomised, controlled trial with trazodone. *Dement Geriatr Cogn Disord* 2004;**17**(4):355-9.
35. Cedarbaum JM, Stambler N, Malta E, et al. The ALSFRS-R: a revised ALS functional rating scale that incorporates assessments of respiratory function. BDNF ALS Study Group (Phase III). *Journal of the neurological sciences* 1999;**169**(1-2):13-21.
36. Kaufmann P, Levy G, Montes J, et al. Excellent inter-rater, intra-rater, and telephone-administered reliability of the ALSFRS-R in a multicenter clinical trial. *Amyotrophic lateral sclerosis : official publication of the World Federation of Neurology Research Group on Motor Neuron Diseases* 2007;**8**(1):42-6.
37. Atassi N, Berry J, Shui A, et al. The PRO-ACT database: design, initial analyses, and predictive features. *Neurology* 2014;**83**(19):1719-25.
38. PRO-ACT Open Access ALS Clinical Trial database Secondary PRO-ACT Open Access ALS Clinical Trial database http://www.alsdatabase.org/.
39. Clinical Trials Facilitation and Coordination Group. Recommendation Paper on the Initiation and Conduct of Complex Clinical Trials. .

For peer review only

FIGURES

Figure legends

Figure 1 Trial overview of MND-SMART

Figure 2 MND-SMART participant timeline. Boxes with red outline indicate study visits which can be conducted by telephone or secure videoconferencing. ALSFRS-R, Amyotrophic Lateral Sclerosis Functional Rating Scale Revised; CARE-MND, Clinical Audit Research Evaluation Motor Neuron Disease; ECAS, Edinburgh Cognitive and Behavioural ALS Screen; ECG, electrocardiogram; FVC, Forced Vital Capacity; HADS, Hospital Anxiety and Depression Scale.

For peer review only

For peer review only - http://bmjopen.bmj.com/site/about/guidelines.xhtml

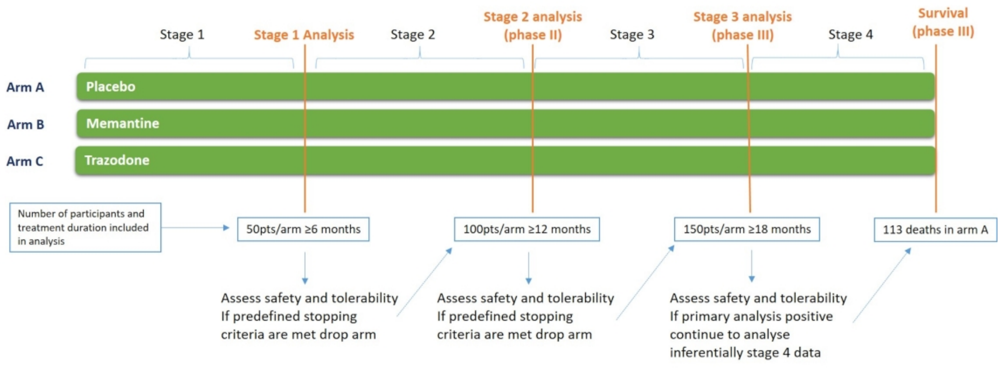

Trial overview of MND-SMART

326x121mm (400 x 400 DPI)

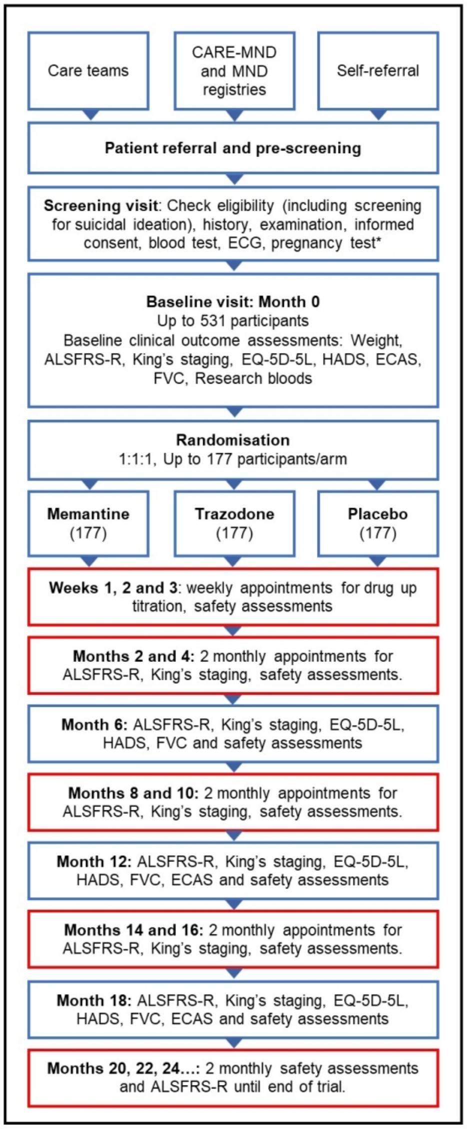

MND-SMART participant timeline. Boxes with red outline indicate study visits which can be conducted by telephone or secure videoconferencing. ALSFRS-R, Amyotrophic Lateral Sclerosis Functional Rating Scale Revised; CARE-MND, Clinical Audit Research Evaluation Motor Neuron Disease; ECAS, Edinburgh Cognitive and Behavioural ALS Screen; ECG, electrocardiogram; FVC, Forced Vital Capacity; HADS, Hospital Anxiety and Depression Scale.

70x150mm (300 x 300 DPI)

STANDARD PROTOCOL ITEMS: RECOMMENDATIONS FOR INTERVENTIONAL TRIALS

SPIRIT 2013 Checklist: Recommended items to address in a clinical trial protocol and related documents*

Section/item	ItemNo	Description
**Administrative information**		
Title	1	Descriptive title identifying the study design, population, interventions, and, if applicable, trial acronym<br>**Page 1**
Trial registration	2a	Trial identifier and registry name. If not yet registered, name of intended registry<br>**Page 2, 7**
	2b	All items from the World Health Organization Trial Registration Data Set<br>https://www.clinicaltrialsregister.eu/ctr-search/trial/2019-000099-41/GB<br>https://www.clinicaltrials.gov/ct2/show/NCT04302870
Protocol version	3	Date and version identifier<br>**Page 14; 24 and 25; Table 5**
Funding	4	Sources and types of financial, material, and other support<br>**Page 16**
Roles and responsibilities	5a	Names, affiliations, and roles of protocol contributors<br>**Page 1, 16**
	5b	Name and contact information for the trial sponsor<br>**Page 15**<br>https://www.clinicaltrialsregister.eu/ctr-search/trial/2019-000099-41/GB
	5c	Role of study sponsor and funders, if any, in study design; collection, management, analysis, and interpretation of data; writing of the report; and the decision to submit the report for publication, including whether they will have ultimate authority over any of these activities<br>**Page 16**

For peer review only

	5d	Composition, roles, and responsibilities of the coordinating centre, steering committee, endpoint adjudication committee, data management team, and other individuals or groups overseeing the trial, if applicable (see Item 21a for data monitoring committee)
**Introduction**		**Page 15**
Background and rationale	6a	Description of research question and justification for undertaking the trial, including summary of relevant studies (published and unpublished) examining benefits and harms for each intervention **Page 5, 6, 9**
	6b	Explanation for choice of comparators **Page 5, 6, 9**
Objectives	7	Specific objectives or hypotheses **Page 6, 7**
Trial design	8	Description of trial design including type of trial (eg, parallel group, crossover, factorial, single group), allocation ratio, and framework (eg, superiority, equivalence, noninferiority, exploratory) **Page 7, 8**
**Methods: Participants, interventions, and outcomes**		
Study setting	9	Description of study settings (eg, community clinic, academic hospital) and list of countries where data will be collected. Reference to where list of study sites can be obtained **Page 8**
Eligibility criteria	10	Inclusion and exclusion criteria for participants. If applicable, eligibility criteria for study centres and individuals who will perform the interventions (eg, surgeons, psychotherapists) **Page 8; Table 2**
Interventions	11a	Interventions for each group with sufficient detail to allow replication, including how and when they will be administered **Page 9; Table 3**
	11b	Criteria for discontinuing or modifying allocated interventions for a given trial participant (eg, drug dose change in response to harms, participant request, or improving/worsening disease) **Page 9**
	11c	Strategies to improve adherence to intervention protocols, and any procedures for monitoring adherence (eg, drug tablet return, laboratory tests) **Page 9**

For peer review only

	11d	Relevant concomitant care and interventions that are permitted or prohibited during the trial<br>**Page 10**
Outcomes	12	Primary, secondary, and other outcomes, including the specific measurement variable (eg, systolic blood pressure), analysis metric (eg, change from baseline, final value, time to event), method of aggregation (eg, median, proportion), and time point for each outcome. Explanation of the clinical relevance of chosen efficacy and harm outcomes is strongly recommended<br>**Page 10, 11, 12, 13**
Participant timeline	13	Time schedule of enrolment, interventions (including any run-ins and washouts), assessments, and visits for participants. A schematic diagram is highly recommended (see Figure)<br>**Page 10; Table 4; Figure 2**
Sample size	14	Estimated number of participants needed to achieve study objectives and how it was determined, including clinical and statistical assumptions supporting any sample size calculations<br>**Page 10, 11**
Recruitment	15	Strategies for achieving adequate participant enrolment to reach target sample size<br>**Page 8**

Methods: Assignment of interventions (for controlled trials)

Allocation:

Sequence generation	16a	Method of generating the allocation sequence (eg, computer-generated random numbers), and list of any factors for stratification. To reduce predictability of a random sequence, details of any planned restriction (eg, blocking) should be provided in a separate document that is unavailable to those who enrol participants or assign interventions<br>**Page 11**
Allocation concealment mechanism	16b	Mechanism of implementing the allocation sequence (eg, central telephone; sequentially numbered, opaque, sealed envelopes), describing any steps to conceal the sequence until interventions are assigned<br>**Page 11**
Implementation	16c	Who will generate the allocation sequence, who will enrol participants, and who will assign participants to interventions<br>**Page 11**

Blinding (masking)	17a	Who will be blinded after assignment to interventions (eg, trial participants, care providers, outcome assessors, data analysts), and how<br>**Page 11**
	17b	If blinded, circumstances under which unblinding is permissible, and procedure for revealing a participant's allocated intervention during the trial<br>**Page 11**
**Methods: Data collection, management, and analysis**		
Data collection methods	18a	Plans for assessment and collection of outcome, baseline, and other trial data, including any related processes to promote data quality (eg, duplicate measurements, training of assessors) and a description of study instruments (eg, questionnaires, laboratory tests) along with their reliability and validity, if known. Reference to where data collection forms can be found, if not in the protocol<br>**Page 10, 12**
	18b	Plans to promote participant retention and complete follow-up, including list of any outcome data to be collected for participants who discontinue or deviate from intervention protocols<br>**Page 7, 13, 14**
Data management	19	Plans for data entry, coding, security, and storage, including any related processes to promote data quality (eg, double data entry; range checks for data values). Reference to where details of data management procedures can be found, if not in the protocol<br>**Page 12**
Statistical methods	20a	Statistical methods for analysing primary and secondary outcomes. Reference to where other details of the statistical analysis plan can be found, if not in the protocol<br>**Page 11**
	20b	Methods for any additional analyses (eg, subgroup and adjusted analyses)<br>**Page 12, 13**
	20c	Definition of analysis population relating to protocol non-adherence (eg, as randomised analysis), and any statistical methods to handle missing data (eg, multiple imputation)<br>**Page 12, 13**
**Methods: Monitoring**		

Data monitoring	21a	Composition of data monitoring committee (DMC); summary of its role and reporting structure; statement of whether it is independent from the sponsor and competing interests; and reference to where further details about its charter can be found, if not in the protocol. Alternatively, an explanation of why a DMC is not needed<br>**Page 13, 15**
	21b	Description of any interim analyses and stopping guidelines, including who will have access to these interim results and make the final decision to terminate the trial<br>**Page 7, 13, 14**
Harms	22	Plans for collecting, assessing, reporting, and managing solicited and spontaneously reported adverse events and other unintended effects of trial interventions or trial conduct<br>**Page 13**
Auditing	23	Frequency and procedures for auditing trial conduct, if any, and whether the process will be independent from investigators and the sponsor<br>**Page 14**
**Ethics and dissemination**		
Research ethics approval	24	Plans for seeking research ethics committee/institutional review board (REC/IRB) approval<br>**Page 14**
Protocol amendments	25	Plans for communicating important protocol modifications (eg, changes to eligibility criteria, outcomes, analyses) to relevant parties (eg, investigators, REC/IRBs, trial participants, trial registries, journals, regulators)<br>**Page 14; Table 5**
Consent or assent	26a	Who will obtain informed consent or assent from potential trial participants or authorised surrogates, and how (see Item 32)<br>**Page 8**
	26b	Additional consent provisions for collection and use of participant data and biological specimens in ancillary studies, if applicable
Confidentiality	27	How personal information about potential and enrolled participants will be collected, shared, and maintained in order to protect confidentiality before, during, and after the trial<br>**Page 14, 15**
Declaration of interests	28	Financial and other competing interests for principal investigators for the overall trial and each study site<br>**Page 16**

For peer review only

Access to data	29	Statement of who will have access to the final trial dataset, and disclosure of contractual agreements that limit such access for investigators<br>**Page 14**
Ancillary and post-trial care	30	Provisions, if any, for ancillary and post-trial care, and for compensation to those who suffer harm from trial participation
Dissemination policy	31a	Plans for investigators and sponsor to communicate trial results to participants, healthcare professionals, the public, and other relevant groups (eg, via publication, reporting in results databases, or other data sharing arrangements), including any publication restrictions<br>**Page 15**
	31b	Authorship eligibility guidelines and any intended use of professional writers
	31c	Plans, if any, for granting public access to the full protocol, participant-level dataset, and statistical code<br>**NA**
**Appendices**		
Informed consent materials	32	Model consent form and other related documentation given to participants and authorised surrogates<br>**Supplementary material**
Biological specimens	33	Plans for collection, laboratory evaluation, and storage of biological specimens for genetic or molecular analysis in the current trial and for future use in ancillary studies, if applicable

*It is strongly recommended that this checklist be read in conjunction with the SPIRIT 2013 Explanation & Elaboration for important clarification on the items. Amendments to the protocol should be tracked and dated. The SPIRIT checklist is copyrighted by the SPIRIT Group under the Creative Commons "Attribution-NonCommercial-NoDerivs 3.0 Unported" license.

For peer review only
