# Supplementary material for: Motor Neuron Disease Systematic Multi-Arm Adaptive Randomised Trial (MND-SMART): a multi-arm, multi-stage, adaptive, platform, phase III randomised, double-blind, placebo-controlled trial of repurposed drugs in motor neuron disease
Source: BMJ Open. 2022 Jul 7;12(7):e064173. doi: 10.1136/bmjopen-2022-064173 (PMC9263927; doi:10.1136/bmjopen-2022-064173)
Supplement: Reviewer comments [file bmjopen-2022-064173.reviewer_comments.pdf]

PEER REVIEW HISTORY

BMJ Open publishes all reviews undertaken for accepted manuscripts. Reviewers are asked to complete a checklist review form (**http://bmjopen.bmj.com/site/about/resources/checklist.pdf**) and are provided with free text boxes to elaborate on their assessment. These free text comments are reproduced below.

ARTICLE DETAILS

**TITLE (PROVISIONAL)**	Motor Neuron Disease Systematic Multi-Arm Adaptive Randomised Trial (MND-SMART): A multi-arm, multi-stage, adaptive, platform, phase III randomised, double-blind, placebo-controlled trial of repurposed drugs in motor neuron disease.
**AUTHORS**	Wong, Charis; Dakin, Rachel; Williamson, Jill; Newton, Judith; Steven, Michelle; Colville, Shuna; Chataway, Jeremy; Swingler, Robert; Parker, Richard; Weir, Christopher; Stallard, Nigel; Parmar, Mahesh; Macleod, Malcolm; Pal, Suvankar; Chandran, Siddharthan

This article was not externally reviewed at BMJ Open. Protocols that have been independently assessed prior to submission to BMJ Open are usually fast-tracked to publication on the grounds that further substantial changes will not be possible. This independent assessment will usually be external, independent review for both a substantial grant award from a non-commercial or government funder and ethics approval.
